# Cortical and Commissural Defects Upon HCF‐1 Loss in *Nkx2.1*‐Derived Embryonic Neurons and Glia

**DOI:** 10.1002/dneu.22704

**Published:** 2019-06-25

**Authors:** Shilpi Minocha, Winship Herr

**Affiliations:** ^1^ Center for Integrative Genomics, Génopode University of Lausanne Lausanne CH‐1015 Switzerland

**Keywords:** Nkx2.1, cortex, anterior commissure, corpus callosum, polymicrogyria, glia, GABAergic neurons

## Abstract

Formation of the cerebral cortex and commissures involves a complex developmental process defined by multiple molecular mechanisms governing proliferation of neuronal and glial precursors, neuronal and glial migration, and patterning events. Failure in any of these processes can lead to malformations. Here, we study the role of HCF‐1 in these processes. HCF‐1 is a conserved metazoan transcriptional co‐regulator long implicated in cell proliferation and more recently in human metabolic disorders and mental retardation. Loss of HCF‐1 in a subset of ventral telencephalic Nkx2.1‐positive progenitors leads to reduced numbers of GABAergic interneurons and glia, owing not to decreased proliferation but rather to increased apoptosis before cell migration. The loss of these cells leads to development of severe commissural and cortical defects in early postnatal mouse brains. These defects include mild and severe structural defects of the corpus callosum and anterior commissure, respectively, and increased folding of the cortex resembling polymicrogyria. Hence, in addition to its well‐established role in cell proliferation, HCF‐1 is important for organ development, here the brain.

## Introduction

Proper development of the cerebral cortex and commissures is achieved by a long and controlled process of proliferation, differentiation, migration, and organization of neuronal and glial cells (Marin and Rubenstein, 2001; Schuurmans and Guillemot, 2002; Mochida and Walsh, 2004; Barkovich *et al.*, 2005; Guillemot *et al.*, 2006; Guerrini and Parrini, 2010). Defects in any of these developmental steps can lead to developmental disorders due to (i) insufficient production of cortical cells or increased cell death, as in microcephaly (Mochida, 2009; Thornton and Woods, 2009; Kaindl *et al.*, 2010), (ii) delayed, excessive, or arrested migration, as in periventricular heterotopia, subcortical band heterotopia, pachygyria, or cobblestone lissencephaly (Francis *et al.*, 2006; Kerjan and Gleeson, 2007; Guerrini and Parrini, 2010), (iii) defective migration of commissural projections owing to improper neuronal/glia positioning and guidance, as in agenesis of corpus callosum, a malformation of the anterior commissure (Silver, 1993; Lindwall *et al.*, 2007; Niquille *et al.*, 2009; 2013; Benadiba *et al.*, 2012; Minocha *et al.*, 2015b), and/or (iv) migration defects combined with defective cortical organization, as in schizencephaly or polymicrogyria (Francis *et al.*, 2006; Barkovich, 2010).

Here, we study the role of the X‐linked *Hcfc1* gene in these processes. *Hcfc1* encodes HCF‐1, a conserved transcriptional co‐regulator that binds to the transcriptional start sites of many genes (Dejosez *et al.*, 2010; Michaud *et al.*, 2013) and associates with both sequence‐specific DNA‐binding proteins (e.g., Myc, E2F1 and E2F4, Thap11/Ronin, ZNF143) and chromatin‐modifying enzymes (e.g., the MLL and Set1 histone H3 Lysine 4 methyltransferases and Sin3 histone deacetylase) (reviewed in (Zargar and Tyagi, 2012); see also (Thomas *et al.*, 2016)). Genetic studies in mammalian cell culture, early mouse embryos, and liver have shown that HCF‐1 is important for multiple aspects of cell proliferation (Goto *et al.*, 1997; Reilly and Herr, 2002; Julien and Herr, 2003; Minocha *et al.*, 2016b), and very early epiblast‐specific embryonic loss of HCF‐1 is lethal before gastrulation (Minocha *et al.*, 2016a; 2016b). Nevertheless, in humans, there are mutations in the *HCFC1* gene that are associated with X‐linked intellectual disability (ID) and cobalamin metabolism; these disorders point toward an important role of HCF‐1 in brain development (Huang *et al.*, 2012; Yu *et al.*, 2013; Gerard *et al.*, 2015; Jolly *et al.*, 2015; Koufaris *et al.*, 2016).

In this study, we investigated the functions of *Hcfc1* in the mouse brain. Conditional loss of HCF‐1 in ventral telencephalic Nkx2.1^+^ progenitors did not appear to affect their proliferation, and yet fewer Nkx2.1‐derived GABAergic interneurons and glia arose upon loss of HCF‐1, owing to increased apoptosis. Reduced migration of GABAergic interneurons and glia was accompanied with corpus callosum defects and abnormal formation of the anterior commissure as well as severe cortical defects that resembled polymicrogyria.

## Materials and Methods

### Mice

All experimental studies have been performed in compliance with the EU and national legislation rules, as advised by the Lemanic Animal Facility Network (Resal), concerning ethical considerations of transportation, housing, strain maintenance, breeding, and experimental use of animals. Mice were housed four to five per cage at 23°C with *ad libitum* food and water access. For staging of embryos, midday of the day of vaginal plug formation was considered as embryonic day 0.5 (E0.5). WT mice maintained in a C57Bl/6 genetic background were used. We used heterozygous *GAD67^_^GFP* knock‐in mice, described in this work as *GAD67‐GFP* mice (Tamamaki *et al.*, 2003). *GAD67^_^GFP* embryos could be recognized by their GFP fluorescence. PCR genotyping of these lines was performed as described previously (Niquille *et al.*, 2009). We used *Hcfc1* ^lox/lox^ (Minocha *et al.*, 2016b), *Nkx2.1*‐*Cre* (Xu *et al.*, 2008), and *GLAST‐Cre:ERT2* (The Jackson Laboratory, Bar Harbor, Maine, USA, Tg(Slc1a3‐cre/ERT)1Nat/J)) (Minocha *et al.*, 2015b) transgenic mice described previously. The reporter *Rosa26R–GFP* mouse line was used to reliably express GFP under the control of the Rosa26 promoter upon Cre‐mediated recombination. The control *Nkx2.1‐Cre^–^/Rosa26‐GFP^+^* and *Hcfc1^lox/Y^/Rosa26‐GFP^+^* did not show any GFP labeling. The control *GLAST‐Cre:ERT2^+^/Rosa26‐GFP^+^* brains did not show any GFP labeling without tamoxifen treatment. For the induction of CreERT, tamoxifen (20 mg ml^−1^, Sigma, St Louis, MO) was dissolved at 37°C in 5 ml corn oil (Sigma) pre‐heated at 42°C for 30 min. A single dose of 4 mg (250–300 μl) was administered to pregnant females.

### Tissue Immunohistochemistry and Histology

Embryos were collected after Caesarean section and quickly killed by decapitation. Their brains were dissected out and fixed by immersion overnight in a solution of 4% paraformaldehyde in 0.1 M of phosphate buffer (pH 7.4) at 4°C. Postnatal mice were profoundly anesthetized and perfused with the same fixative and their brains post‐fixed for 4 h. Brains were cryoprotected in a solution of 30% sucrose in 0.1 M phosphate buffer (pH 7.4), frozen and cut in 50‐μm‐thick coronal sections for fluorescence immunostaining. For diaminobenzidine (DAB) immunostaining, the brain tissues were paraffin‐embedded and sectioned into 8 μm thick sections using a MICROM HM325 microtome. For each immunostaining, we made use of several mice (between three and six) for both control and mutant strains analyzed. The method for paraffin‐ and cryo‐sections staining was as follows:

- DAB and fluorescence immunostaining:
  The paraffin‐embedded sections were first (i) deparaffinized in xylene, (ii) rehydrated through graded alcohol washes, and (iii) rinsed twice with PBS. For DAB immunostaining, endogenous peroxidase activity was quenched at this stage with 6% hydrogen peroxide in methanol for 10 min and rapidly washed once with H_2_O. Subsequently antigens were revealed by heating in a 750 W microwave oven until boiling for approximately 10 min in citrate buffer (10 mM, pH 6.0), allowed to slowly cool to 4°C, washed twice with PBS, and then blocked for 30 min with 2% normal goat serum (NGS) (Sigma‐Aldrich, cat. # G9023) in PBS at room temperature (RT).
  For fluorescence immunostaining, cryosections were (i) rinsed thrice with PBS, (ii) rinsed thrice with PBS containing 0.3% Triton X‐100, and (iii) blocked for 1 hr with 2% NGS in PBS solution with 0.3% TritonX‐100 at RT.
  After blocking, primary immunostaining was performed by the incubation of the slices with specific primary antibody (see below) diluted in 2% NGS overnight at 4°C followed by three washes with PBS.
  For DAB immunostaining, the primary antibodies were detected by incubating the sections for 30 min with anti‐mouse (Dako cat. # K4000) or anti‐rabbit (Dako cat. # K4002) horseradish peroxidase (HRP) secondary antibody. Visualization was performed with DAB substrate (Dako cat. # K3468) before being counterstained with Mayer’s hematoxylin.
  For fluorescence immunostaining, incubation with the appropriate secondary antibody (see below) was for 30 min in the dark at RT for paraffin sections and 90 min in the dark at RT for cryo‐sections, followed by (i) three PBS washes, (ii) counterstaining with 4',6‐diamidino‐2‐phenylindole (DAPI) (Sigma‐Aldrich, CAS # 28718‐90‐3), (iii) two PBS washes, and (iv) embedding with Mowiol mounting medium (Sigma‐Aldrich, CAS # 9002‐89‐5).
  The DAB‐stained sections were subsequently imaged using an AxioImager M1 microscope with AxioCam MRm monochrome and AxioCam MRc color cameras (Carl Zeiss AG, Oberkochen, Germany), or a Zeiss CLSM 710 spectral confocal laser scanning microscope. Images were processed using AxioVision 4.8.2 (Carl Zeiss AG, Oberkochen, Germany). Fluorescent immunostained sections were imaged using confocal microscopes (Zeiss LSM 510 or Zeiss LSM 710). Z‐stacks were acquired for each coronal section in a multitrack mode avoiding crosstalk. All three‐dimensional (3D) Z‐stack reconstructions and image processing were performed using Imaris 8.2 (Bitplane Inc.) software. To create real 3D data sets, we used the mode “Surpass.” Figures were processed in Adobe Photoshop CS6, and schematic illustrations were produced using Adobe Illustrator CS6.
  The primary antibodies used were: rat anti‐Ki67 (1:60; eBioscience), anti‐Ctip2 (1:500; Abcam), and anti‐L1 (1:200; Chemicon) antibodies; rabbit anti‐HCF‐1 (1:1000, H12; (Wilson *et al.*, 1993)), anti‐Cux1 (1:200; Santacruz), anti‐Nkx2.1 (1:2,000; Biopat), anti‐GFAP (1:500; DAKO), anti‐Calbindin (1:2500; Swant), and anti‐Calretinin (1:2000; Swant) antibodies; chicken anti‐GFP (1:500; Aves) antibody; and mouse anti‐SatB2 (1:500; gift from V. Tarabykin) and anti‐GFAP (1:500; Chemicon) antibodies.
  The secondary antibodies used were: goat anti‐rabbit Alexa 488 (1:400; Molecular Probes cat. # A11034), goat anti‐mouse Alexa 568 (1:500; Molecular Probes cat. # A11019), goat anti‐rabbit Alexa 568 (1:1000; Molecular Probes cat. # A21069), goat anti‐mouse Alexa 488 (1:400; Molecular Probes cat. # A11029), donkey anti‐mouse Alexa 594 (1:500; Molecular Probes cat. # A11005), and goat anti‐mouse Alexa 635 (1:300; Molecular Probes cat. # A31575) antibodies.
- Colorations:
*Nissl staining*
  Standard Nissl staining was performed on both deparaffinized and rehydrated paraffin sections and cryo‐sections as described (Paul *et al.*, 2008).
*Hematoxylin and eosin staining*
  Standard hematoxylin and eosin staining were performed on deparaffinized and rehydrated lung sections (Fischer *et al.*, 2008).

### TUNEL Assay

Terminal deoxynucleotidyltransferase‐mediated dUTP‐biotin nick end labeling (TUNEL) was performed on brain sections with the *in situ* cell death detection kit (Roche Applied Science, cat. # 11684795910), according to the manufacturer's directions.

### Immunoblotting

For immunoblotting, approximately 100 mg of brain tissue was homogenized in RIPA buffer (50 mM Tris‐HCl ph 7.4, 150 mM NaCl, 1 mM EDTA, 0.2% sodium deoxycholate, 1 mM DTT, 1 mM PMSF, and 1% Triton X) containing protease inhibitors (Roche). Samples (10–20 μg) were boiled for 5 min before PAGE and transfer to nitrocellulose membrane. Membranes were blocked for 60 min with 5 ml of LI‐COR blocking buffer, incubated with primary antibody in 50% LI‐COR blocking buffer and 50% PBST (PBS containing 0.1% Tween 20) overnight at 4°C, washed three times and incubated with secondary antibody (dilution 1:10,000) for 30 min at RT. The membranes were washed three times and scanned with an Odyssey infrared imager (LI‐COR).

The primary antibodies were as follows and used at the following dilutions: rabbit anti‐HCF‐1 (1:1000, H12, (Wilson *et al.*, 1993)) and anti‐actin (1:5000, Sigma‐Aldrich) antibodies. The secondary antibodies used were: donkey anti‐rabbit IRDye 680RD (1∶10,000; LI‐COR Inc. cat. # 926‐68073), donkey anti‐mouse IRDye 680RD (1∶10,000; LI‐COR Inc. cat. # 926‐68072), donkey anti‐rabbit IRDye 800CW (1∶10,000; LI‐COR Inc. cat. # 926‐32213), and donkey anti‐mouse IRDye 800CW (1∶10,000; LI‐COR Inc. cat. # 926‐32212).

### Quantitation and Statistical Analyses

For each analysis, all cells in a representative field of either 8 µm‐thick paraffin sections or 50 µm cryo‐sections were counted. Mutant embryos/pups were always compared with controls originating from the same litter. In each case, entire fields acquired at same magnification were quantitated. For all analyses, values from at least three independent experiments were first tested for normality and the variance of independent populations were tested for equality. Student’s *t‐test* was performed using the R package (www.r-project.org). To show the degree of significance for quantitation included in the study, we added the number of asterisks based on the following standard *P*‐value criteria: ***P < 0.001; **P < 0.01; *P ≤ 0.05.

### Atlas and Nomenclature

The neuroanatomical nomenclature is based on the “Atlas of the prenatal mouse brain” (Schambra *et al.*, 1991).

## Results

### Broad *Hcfc1* Expression in Mouse Brain

**Figure 1:**
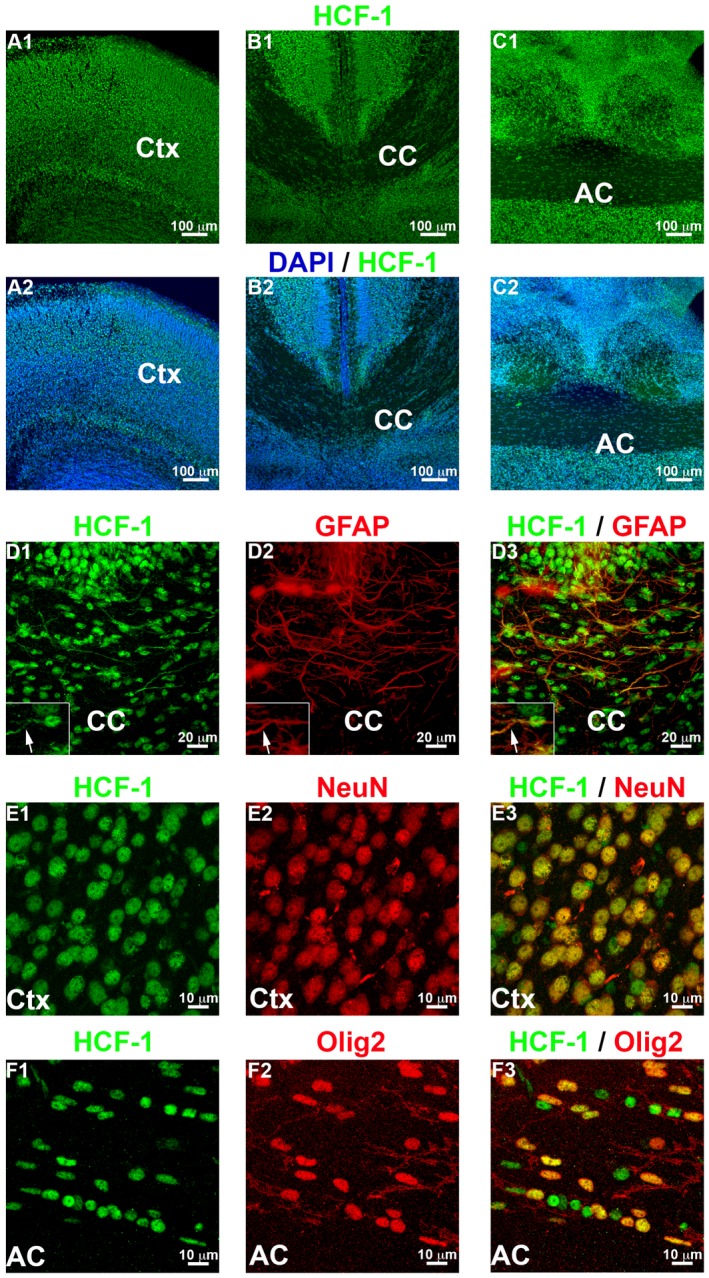
*Hcfc1* is broadly expressed in the mouse postnatal brain. (A–C) Immunofluorescence analysis of cryo‐sections from wildtype *C57BL6* brains at P0 stained with DAPI (blue) and antibody against HCF‐1 (green). Staining with only anti‐HCF‐1 (A1, B1, and C1) and colocalization between anti‐HCF‐1 and DAPI staining (A2, B2, and C2) is shown in cortex (Ctx; A), corpus callosum (CC; B), and anterior commissure (AC; C) region. (D) Immunofluorescence analysis of cryo‐sections from wildtype *C57BL6* brains at P0 stained with antibodies against HCF‐1 (green) and astroglial marker, glial fibrillary acidic protein (GFAP; red). The inset shows a GFAP‐positive glia at higher magnification. (E) Immunofluorescence analysis of cryo‐sections from wildtype *C57BL6* brains at P0 stained with antibodies against HCF‐1 (green) and neuronal marker, NeuN (red). (F) Immunofluorescence analysis of cryo‐sections from wildtype *C57BL6* brains at P0 stained with antibodies against HCF‐1 (green) and oligodendrocyte marker, Olig2 (red). Scale bars are indicated in the figure.

Before investigating the effects of loss of *Hcfc1* function in brain cells, we assayed its expression in the developing mouse brain. The human *HCFC1* and mouse *Hcfc1* genes are highly expressed in actively dividing tissue culture cells, and in embryonic and placental tissues and in adult tissues (Wilson *et al.*, 1995; Frattini *et al.*, 1996; Kristie, 1997; Huang *et al.*, 2012; Minocha *et al.*, 2016b). Previously, using a well‐characterized antibody generating little to no non‐specific reactivity (H12), we have shown that HCF‐1 is ubiquitous and predominantly nuclear in E6.5‐to‐E12.5 embryos, postnatal day 0 (P0) brains, and 10‐week‐old young adult brains (Minocha *et al.*, 2016b). Here, we further investigated in detail the cellular and subcellular localization of HCF‐1 in the early mouse brain by immunostaining wild‐type C57BL/6 postnatal day 0 (P0) brains. We focused on the cortex (Ctx), corpus callosum (CC), and anterior commissure (AC) — brain regions affected by the *Nkx2.1‐Cre*‐engineered loss of HCF‐1 described here. In these three regions, HCF‐1 was found to be ubiquitous (Fig. 1A–C) and predominantly nuclear in the GFAP‐positive astroglia (Fig. 1D), NeuN‐positive neurons (Fig. 1E), and Olig2‐positive oligodendrocytes (Fig. 1F), with astroglial‐cell processes showing additional relatively faint staining (Fig. 1D, see arrow). Immunoblotting analysis of postnatal and adult mouse brains ranging from P1 to 1.2 years old demonstrated continued expression of *Hcfc1* into adulthood (Supp. Fig. 1A1) (Minocha *et al.*, 2016b), but with a progressive reduction in relative HCF‐1 protein levels with age (Supp. Fig. 1A2). Such a broad and long‐term expression profile of *Hcfc1* suggests that HCF‐1 plays roles in both young and adult mouse brains.

### Induced Loss of HCF‐1 in *Nkx2.1*‐Positive Cells in the Ventral Telencephalon

We wished to probe such roles for HCF‐1 in brain development and decided to use *Nkx2.1‐Cre* mediated *Hcfc1*‐gene inactivation for this purpose. In the embryonic brain, the *Nkx2.1* homeobox gene is expressed in a region of the forebrain that develops into the ventral telencephalon which includes the medial ganglionic eminence (MGE), the anterior entopeduncular area (AEP), the anterior preoptic area (POA), the septum (SEP) and parts of the amygdala (Lazzaro *et al.*, 1991; Kimura *et al.*, 1996; Sussel *et al.*, 1999; Puelles *et al.*, 2000; Flames *et al.*, 2007). In the MGE, *Nkx2.1* is expressed in the rapidly dividing ventricular (VZ) and sub‐ventricular zones (SVZ) as well as the mantle zone comprised of migratory cells (Sussel *et al.*, 1999), and is important for the generation of both neuronal (GABAergic interneurons) and glial (astroglia and oligodendrocytes) cell types (Sussel *et al.*, 1999; Anderson *et al.*, 2001; Corbin *et al.*, 2001; Marin and Rubenstein, 2001; Kessaris *et al.*, 2006; Minocha *et al.*, 2015a; 2015b). These cells ensure proper cortical development and function, and commissure formation (Wonders and Anderson, 2006; Lindwall *et al.*, 2007; Minocha *et al.*, 2015b). Importantly, as shown in Figure 1, *Hcfc1* is highly expressed in these cell types.

We induced conditional deletion of the *Hcfc1* gene in these cell types by crossing males heterozygous for an *Nkx2.1‐Cre* transgene (referred to as *Nkx2.1‐Cre* ^+^) (Xu *et al.*, 2008) with *Hcfc1* ^lox/lox^ females (Minocha *et al.*, 2016b). The X‐linked *Hcfc1* ^lox^ allele contains two loxP sites, one in intron 1 and another in intron 3 that undergo recombination in the presence of Cre recombinase, deleting exons 2 and 3 to generate the conditional knockout (cKO) allele encoding a highly truncated 66 amino acid long N‐terminal HCF‐1 protein (Minocha *et al.*, 2016b). Thus, hemizygous *Hcfc1^lox/Y^* males carrying the *Nkx2.1‐Cre* ^+^ allele were expected to generate a complete embryonic *Nkx2.1*‐specific knock out. The resulting male progeny of the aforementioned cross generated the control strain *Nkx2.1‐Cre* ^+^; *Hcfc1^+/Y^* and the *Nkx2.1‐Cre*‐induced knockout strain *Nkx2.1‐Cre^+^*; *Hcfc1^lox/Y^*. The *Nkx2.1‐Cre^+^*; *Hcfc1^lox/Y^* postnatal mice appeared to be ill, probably owing to improper lung formation (compare Supp. Fig. 2A to B and C; see [Lazzaro *et al.*, 1991]), and often suffered from maternal cannibalism or died during early postnatal ages around P5.

To follow the generation of *Nkx2.1‐Cre*‐induced conditional knockout allele, we used a Cre‐recombination inducible *Rosa‐GFP* reporter, which can mark *Nkx2.1‐Cre‐*expressing cells and hence identify likely conditional knockout allele‐containing cells. As expected, the control *Rosa‐GFP^+^*; *Hcfc1^lox/Y^* embryos lacking the *Nkx2.1‐Cre* allele did not show specific GFP labeling (Supp. Fig. 3A). As *Nkx2.*1 expression can be seen as early as embryonic (E) day 10.5 (Sussel *et al.*, 1999), we began by investigating the generation of conditional knockout allele‐containing cells in embryonic *Nkx2.1‐Cre^+^*; *Rosa‐GFP^+^*; *Hcfc1^lox/Y^* brains at E12.5 and E14.5. For clarity, here, the control strain is referred to as *Nkx2.1‐Cre* ^+^; *Rosa‐GFP^+^*; *Hcfc1^+/Y^* and the *Nkx2.1‐Cre*‐induced knockout strain is referred to as *Nkx2.1‐Cre^+^*; *Rosa‐GFP^+^*; *Hcfc1^lox/Y^*.

**Figure 2:**
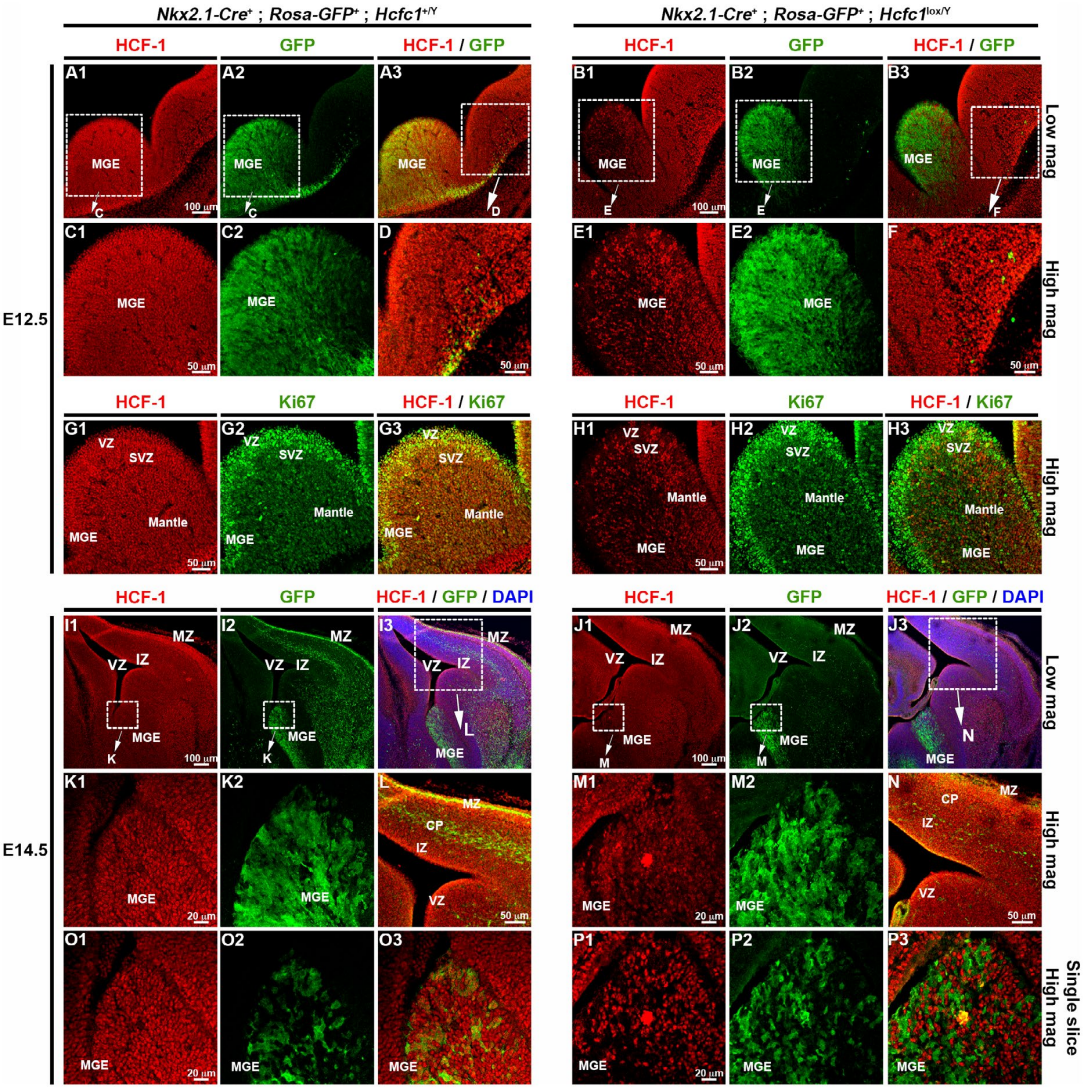
*Nkx2.1‐Cre* mediated loss of HCF‐1 leads to decreased generation of ventral telencephalic cells. Immunofluorescence analysis of cryo‐sections from control *Nkx2.1‐Cre* ^+^; *Rosa‐GFP* ^+^; *Hcfc1^+/Y^* (A, C, D, I, K, and O) and knockout *Nkx2.1‐Cre* ^+^; *Rosa‐GFP* ^+^; *Hcfc1^lox/Y^* (B, E, F, J, M, and P) embryonic brains at E12.5 (A‐to‐F) and E14.5 (I‐to‐P) stained with antibodies against HCF‐1 (red) and GFP (green). The boxed region of medial ganglionic eminence (MGE) shown in A, B, I, and J is shown as higher magnification in C, E, K, and M, respectively. The HCF‐1 (red) and GFP (green) staining in boxed region in A3, B3, I3, and J3 is shown at higher magnification in D, F, L, and N, respectively. A single layer from the confocal stack shown in K and M is shown in O and P, respectively. (G‐H) Immunofluorescence analysis of cryo‐sections from control *Nkx2.1‐Cre* ^+^; *Rosa‐GFP* ^+^; *Hcfc1^+/Y^* (G) and knockout *Nkx2.1‐Cre* ^+^; *Rosa‐GFP* ^+^; *Hcfc1^lox/Y^* (H) embryonic brains at E12.5 stained with antibodies against HCF‐1 (red) and proliferation marker, Ki67 (green). CP, cortical plate; IZ, intermediate zone; MZ, marginal zone; SVZ, sub‐ventricular zone; VZ, ventricular zone. Scale bars are indicated in the figure.

**Figure 3:**
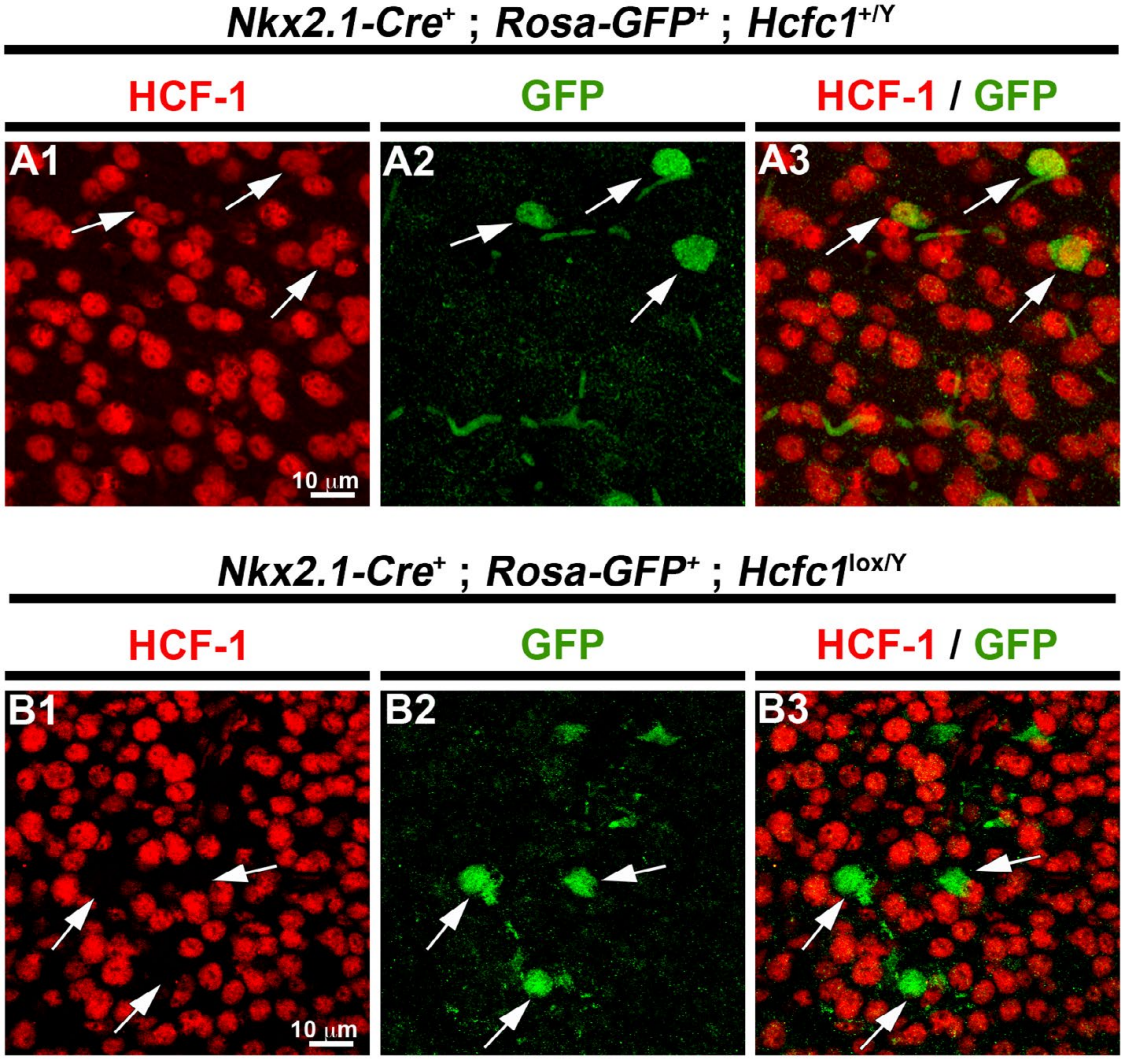
The *Nkx2.1‐Cre* mediated recombination inducible *Rosa‐GFP* reporter faithfully identifies conditional knockout allele‐containing cells. Immunofluorescence analysis of cryo‐sections from control *Nkx2.1‐Cre* ^+^; *Rosa‐GFP* ^+^; *Hcfc1^+/Y^* (A) and knockout *Nkx2.1‐Cre* ^+^; *Rosa‐GFP* ^+^; *Hcfc1^lox/Y^* (B) embryonic brains at E14.5 stained with antibodies against HCF‐1 (red) and GFP (green). The arrows point to *Hcfc1* ^+^ allele‐containing cells that are GFP‐positive and HCF‐1‐positive (A) and *Hcfc1^lox^* allele‐containing cells that are GFP‐positive and HCF‐1‐negative (B), respectively. Scale bars are indicated in the figure.

Similar to the control *Nkx2.1‐Cre^+^*; *Rosa‐GFP^+^*; *Hcfc1^+/Y^* embryos, the *Nkx2.1‐Cre^+^*; *Rosa‐GFP^+^*; *Hcfc1^lox/Y^* embryos displayed temporally and spatially specific *Nkx2.1‐Cre*‐mediated recombination activity, demonstrated by the presence of GFP only in the Nkx2.1‐derived zones such as the MGE and POA (Fig. 2A, B, I and J; Supp. Fig. 3B–C). Coherently, the GFP‐positive cells were found to be HCF‐1‐positive in control *Nkx2.1‐Cre^+^*; *Rosa‐GFP^+^*; *Hcfc1^+/Y^* embryos and HCF‐1‐negative in *Nkx2.1‐Cre^+^*; *Rosa‐GFP^+^*; *Hcfc1^lox/Y^* embryos at E12.5 (compare Fig. 2C and E) and at E14.5 (compare Fig. 2K and M); compare also the single confocal layers shown in Figure 2O and P. The GFP‐positive cells continued to be HCF‐1‐positive in control *Nkx2.1‐Cre^+^*; *Rosa‐GFP^+^*; *Hcfc1^+/Y^* embryos (Fig. 3A) and HCF‐1‐negative in *Nkx2.1‐Cre^+^*; *Rosa‐GFP^+^*; *Hcfc1^lox/Y^* embryos (Fig. 3B) after exiting the MGE region and migrating to the cortex. We note, however, that GFP‐negative cells remained and that in *Nkx2.1‐Cre^+^*; *Rosa‐GFP^+^*; *Hcfc1^lox/Y^* embryos these were HCF‐1 positive, indicating that the *Nkx2.1‐Cre* transgene was not active in all cells (Supp. Fig. 3D). Nevertheless, the very high correspondence between GFP‐positive and HCF‐1‐negative cells (Supp. Fig. 3E) — owing to the cell autonomous function of HCF‐1 — allowed us to follow GFP fluorescence as a marker for HCF‐1‐negative cells.

### Paucity of HCF‐1 Negative Cells in the Developing Cortical Region

**Figure 4:**
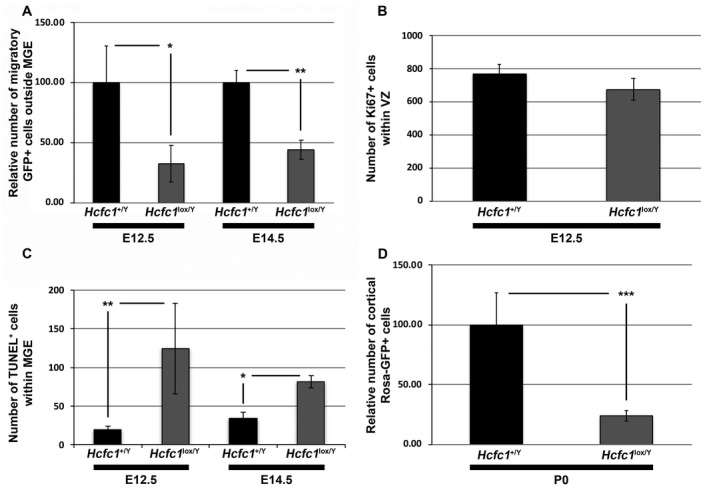
*Nkx2.1‐Cre* mediated loss of HCF‐1 causes decreased migration and increased cell death of ventral telencephalic cells. (A) Graph showing the relative number of GFP^+^ migratory cells outside MGE region in control *Nkx2.1‐Cre^+^*; *Rosa‐GFP* ^+^; *Hcfc1^+/Y^* (n = 2 at E12.5; n = 2 at E14.5; shown as *Hcfc1^+/Y^*) and mutant *Nkx2.1‐Cre* ^+^; *Rosa‐GFP* ^+^; *Hcfc1^lox/Y^* (n = 2 at E12.5; n = 3 at E14.5; shown as *Hcfc1^lox/Y^*) brains at E12.5 and E14.5. The number of GFP^+^ migratory cells outside MGE region in *Nkx2.1‐Cre^+^*; *Rosa‐GFP* ^+^; *Hcfc1^+/Y^* control brains was calculated, and the mean value was set as 100%. The percentage of GFP^+^ migratory cells outside MGE region in *Nkx2.1‐Cre* ^+^; *Rosa‐GFP* ^+^; *Hcfc1^lox/Y^* mutant brains was then calculated relative to the value in control sections. The difference between relative percentage of GFP^+^ migratory cells outside MGE region in control *Nkx2.1‐Cre^+^*; *Rosa‐GFP* ^+^; *Hcfc1^+/Y^* and knockout *Nkx2.1‐Cre* ^+^; *Rosa‐GFP* ^+^; *Hcfc1^lox/Y^* brains at E12.5 was significant (*P*‐value 0.05). The difference between relative percentage of GFP^+^ migratory cells outside MGE region in control *Nkx2.1‐Cre^+^*; *Rosa‐GFP* ^+^; *Hcfc1^+/Y^* and knockout *Nkx2.1‐Cre* ^+^; *Rosa‐GFP* ^+^; *Hcfc1^lox^* ^/Y^ brains at E14.5 was significant (*P*‐value 0.003). (B) Graph showing the number of Ki67^+^ cells in ventricular zone (VZ) of control *Nkx2.1‐Cre^+^*; *Rosa‐GFP* ^+^; *Hcfc1^+/Y^* (n = 3; shown as *Hcfc1^+/Y^*) and knockout *Nkx2.1‐Cre* ^+^; *Rosa‐GFP* ^+^; *Hcfc1^lox/Y^* (n = 2; shown as *Hcfc1^lox/Y^*) brains at E12.5. The difference between number of Ki67^+^ cells in control *Nkx2.1‐Cre^+^*; *Rosa‐GFP* ^+^; *Hcfc1^+/Y^* and knockout *Nkx2.1‐Cre* ^+^; *Rosa‐GFP* ^+^; *Hcfc1^lox/Y^* brains was not significant. (C) Graph showing the number of TUNEL^+^ cells in control *Nkx2.1‐Cre^+^*; *Rosa‐GFP* ^+^; *Hcfc1^+/Y^* (n = 3 at E12.5; n = 2 at E14.5; shown as *Hcfc1^+/Y^*) and knockout *Nkx2.1‐Cre* ^+^; *Rosa‐GFP* ^+^; *Hcfc1^lox/Y^* (n = 5 at E12.5; n = 3 at E14.5; shown as *Hcfc1^lox/Y^*) brains at E12.5 and E14.5. The difference between number of TUNEL^+^ cells in control *Nkx2.1‐Cre^+^*; *Rosa‐GFP* ^+^; *Hcfc1^+/Y^* and knockout *Nkx2.1‐Cre* ^+^; *Rosa‐GFP* ^+^; *Hcfc1^lox/Y^* brains at E12.5 was significant (*P*‐value 0.008). The difference between number of TUNEL^+^ cells in control *Nkx2.1‐Cre^+^*; *Rosa‐GFP* ^+^; *Hcfc1^+/Y^* and knockout *Nkx2.1‐Cre* ^+^; *Rosa‐GFP* ^+^; *Hcfc1^lox/Y^* brains at E14.5 was significant (*P*‐value 0.02). (D) Graph showing the relative number of GFP^+^ cells in cortices of control *Nkx2.1‐Cre^+^*; *Rosa‐GFP* ^+^; *Hcfc1^+/Y^* (n = 3; shown as *Hcfc1^+/Y^*) and knockout *Nkx2.1‐Cre* ^+^; *Rosa‐GFP* ^+^; *Hcfc1^lox/Y^* (n = 3; shown as *Hcfc1^lox/Y^*) brains at P0. The number of cortical GFP^+^ cells in *Nkx2.1‐Cre^+^*; *Rosa‐GFP* ^+^; *Hcfc1^+/Y^* control brains was calculated, and the mean value was set as 100%. The percentage of cortical GFP^+^ cells in *Nkx2.1‐Cre* ^+^; *Rosa‐GFP* ^+^; *Hcfc1^lox/Y^* mutant brains was then calculated relative to the value in control sections. The difference between relative percentage of cortical GFP^+^ cells in control *Nkx2.1‐Cre^+^*; *Rosa‐GFP* ^+^; *Hcfc1^+/Y^* and knockout *Nkx2.1‐Cre* ^+^; *Rosa‐GFP* ^+^; *Hcfc1^lox/Y^* brains was significant (*P*‐value 0.001).

With the induction of loss of HCF‐1 in the MGE, we noted that the number of GFP‐positive (i.e., HCF‐1‐negative) cells in the developing cortical region appeared to be reduced in *Nkx2.1‐Cre^+^*; *Rosa26‐GFP^+^*; *Hcfc1^lox/Y^* embryos compared to control *Nkx2.1‐Cre^+^*; *Rosa26‐GFP^+^*; *Hcfc1^+/Y^* embryos, both at E12.5 (compare Fig. 2A to B; also see higher magnification in Fig. 2D and F) and E14.5 (compare Fig. 2I to J; also see higher magnification in Fig. 2L and N). This reduction in GFP‐positive cells probably reflects a paucity of HCF‐1‐negative migratory cells in the developing cortex. Quantification of GFP‐positive cells showed a 70% and 55% decrease at E12.5 and E14.5, respectively, in *Nkx2.1‐Cre^+^*; *Rosa‐GFP^+^*; *Hcfc1^lox/Y^* vs. control *Nkx2.1‐Cre^+^*; *Rosa26‐GFP^+^*; *Hcfc1^+/Y^* embryos (Fig. 4A).

### Increased Apoptosis in the Ventral Telencephalon after Induction of HCF‐1 Loss

The decrease in proportion of GFP‐positive cells in the developing cortex could be attributed to either a reduction in HCF‐1 negative cells, either owing to defective proliferation or increased cell death, or a defect in migration. We addressed the former possibility here. To investigate a defect in cell proliferation, we performed immunostaining for the cell proliferation marker Ki67. At E12.5, no statistically significant difference in the percentage of Ki67‐positive cells was found between the VZ of control *Nkx2.1‐Cre^+^; Rosa26‐GFP^+^*; *Hcfc1^+/Y^* and *Nkx2.1‐Cre^+^; Rosa26‐GFP^+^; Hcfc1^lox/Y^* embryos (compare Fig. 2G to H; see quantitation Fig. 4B). Indeed, most HCF‐1‐negative cells were Ki67‐positive consistent with active or recent proliferation. Furthermore, Ki67‐positive cells appeared to be evenly distributed in the MGE of *Nkx2.1‐Cre^+^; Rosa26‐GFP^+^; Hcfc1^lox/Y^* embryos (Fig. 2H). Similar results were also observed at E14.5 (data not shown). Thus, although HCF‐1 has been implicated in cell proliferation in vitro (Goto *et al.*, 1997) and in liver regeneration (Minocha *et al.*, 2016b), these results indicate that HCF‐1 is not required for proliferation of MGE neuronal and glial progenitor cells in the developing embryonic mouse brain.

**Figure 5:**
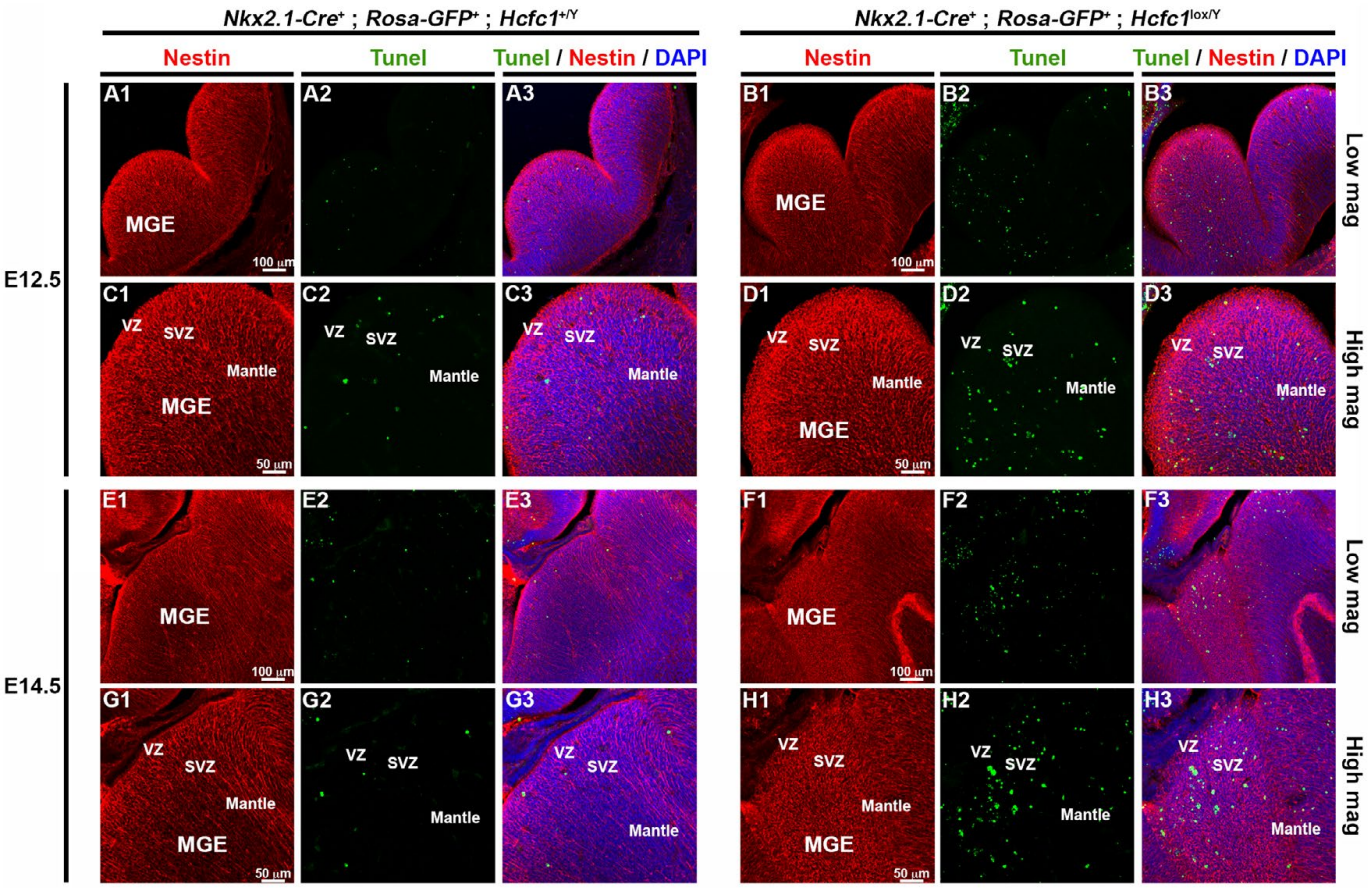
*Hcfc1*‐conditional knockout embryonic brains display increased incidence of cell death. TUNEL assay was performed on cryo‐sections from control *Nkx2.1‐Cre* ^+^; *Rosa‐GFP* ^+^; *Hcfc1^+/Y^* (A, C, E, and G) and knockout *Nkx2.1‐Cre* ^+^; *Rosa‐GFP* ^+^; *Hcfc1^lox/Y^* (B, D, F, and H) embryonic brains at E12.5 (A‐to‐D) and E14.5 (E‐to‐H) co‐stained with DAPI (blue) and antibody against Nestin (red). TUNEL‐positive apoptotic cells are shown in green. The medial ganglionic eminence (MGE) region shown in A, B, E, and F is shown as higher magnification in C, D, G, and H, respectively. SVZ, sub‐ventricular zone; VZ, ventricular zone. Scale bars are indicated in the figure.

Next, we assayed for the possibility of increased cell death in the ventral telencephalon of *Nkx2.1‐Cre^+^; Rosa26‐GFP^+^; Hcfc1^lox/Y^* vs. control embryos. Indeed, TUNEL assays revealed a significantly increased number of apoptotic cells, specifically in the SVZ and the mantle zone, both at E12.5 (compare Fig. 5A and C to B and D, respectively) and E14.5 (compare Fig. 5E and G to F and H, respectively) (see quantification in Fig. 4C). Thus, the reduced number of GFP‐positive—and hence HCF‐1‐negative cells — in the developing cortex (see Figs. 2 and 4A) is likely due to an increased incidence of cell death as opposed to a direct effect on cell proliferation.

### Postnatal Brains Display a Severe Reduction of Interneurons and Glia Upon Induced HCF‐1 Loss

**Figure 6:**
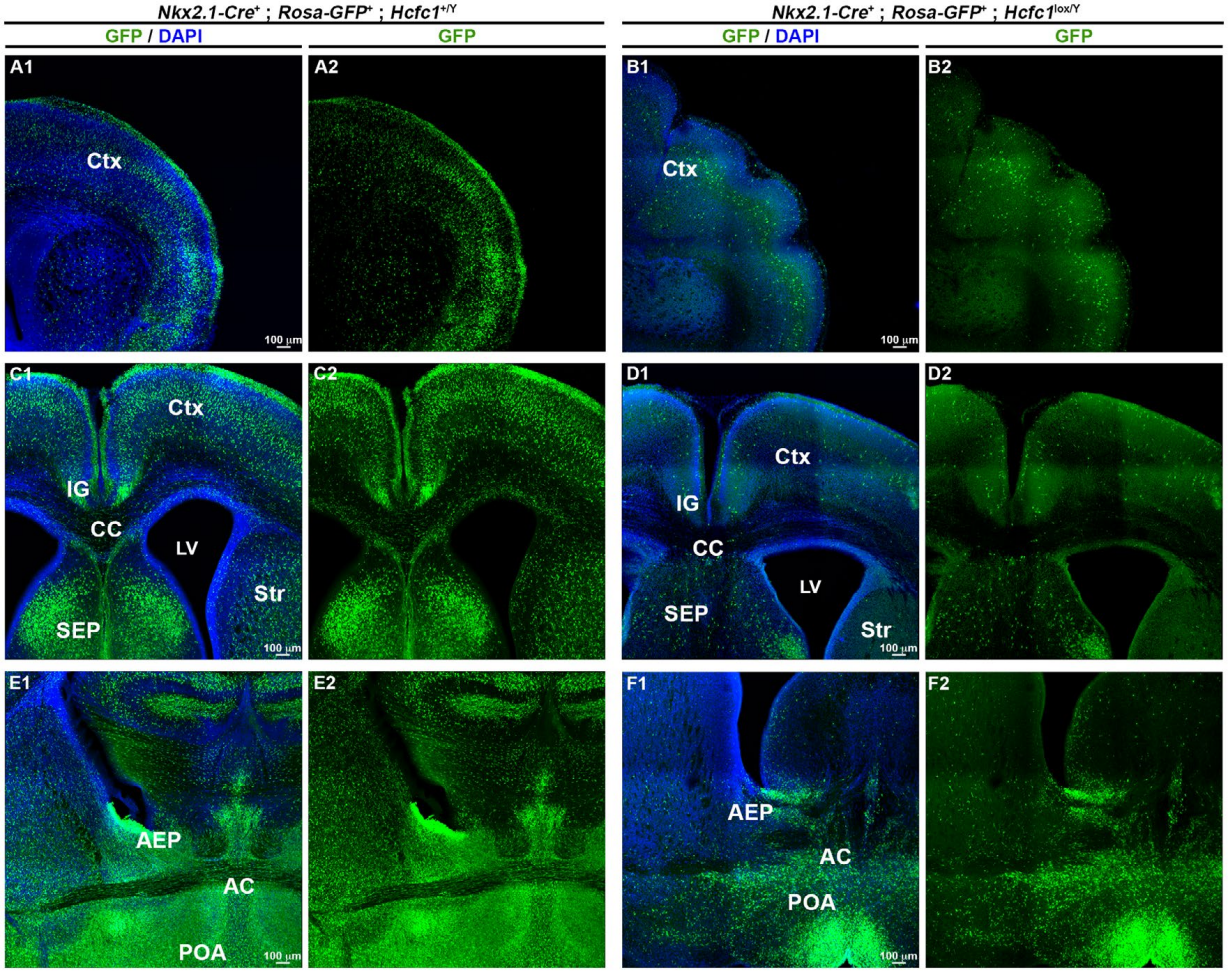
*Nkx2.1‐Cre* mediated loss of HCF‐1 leads to decreased presence of *Nkx2.1*‐derived cells in the postnatal brains. Immunofluorescence analysis of cryo‐sections from control *Nkx2.1‐Cre* ^+^; *Rosa‐GFP* ^+^; *Hcfc1^+/Y^* (A, C, and E) and knockout *Nkx2.1‐Cre* ^+^; *Rosa‐GFP* ^+^; *Hcfc1^lox/Y^* (B, D, and F) brains at P0 stained with DAPI (blue) and antibody against GFP (green). Both anti‐GFP and DAPI staining (A2, B2, and C2) and only anti‐GFP staining (A1, B1, and C1) is shown in cortex (Ctx; A and B), corpus callosum (CC) and surrounding region (C and D), and anterior commissure (AC) and surrounding region (E and F). The panels show confocal images obtained by tiling and stitching nine adjacent regions, acquired with 10x objective, together to display larger areas. AEP, anterior endopenducular area; IG, indusium griseum; LV, lateral ventricles, SEP, septum; Str, striatum; POA, anterior preoptic area. Scale bars are indicated in the figure.

To determine the effects of the aforementioned loss of Nkx2.1‐lineage cells owing to the loss of HCF‐1 on brain development, we turned to postnatal mice. Because, however, postnatal *Nkx2.1‐Cre^+^*; *Hcfc1^lox/Y^* mice are ill, (Supp. Fig. 2), and because they suffered from maternal cannibalism, we studied brains from early postnatal (i.e., neonatal) P0 and P1 pups. Consistent with the loss of HCF‐1‐negative cells during embryonic development, as shown in Figure 6, throughout the brain there were many fewer (approximately 75% less) GFP‐positive cells in knockout *Nkx2.1‐Cre^+^*; *Rosa26‐GFP^+^*; *Hcfc1^lox/Y^* vs. control *Nkx2.1‐Cre^+^*; *Rosa26‐GFP^+^*; *Hcfc1^+/Y^* brains. Regions affected included the entire cortical region (compare Fig. 6A to B; see quantitation Fig. 4D), the corpus callosum (CC), striatum (Str), indusium griseum (IG), and septum (SEP) (compare Fig. 6C to D), and anterior commissure (AC) and surrounding areas including the anterior entopeduncular area (AEP) and anterior preoptic area (POA) (compare Fig. 6E to F).

As *Nkx2.1*‐lineage cells develop into both neurons (GABAergic interneurons) and glia (astroglia and oligodendrocytes), we next determined which, if not both, of these two sublineages are affected by loss of HCF‐1. Firstly, to identify globally the *Nkx2.1*‐derived GABAergic interneuron population, we used the *Gad1 (Glutamate decarboxylase 1*) gene (encoding the GAD67 protein), as *Gad1‐GFP* knock‐in reporter mice where GAD67^+^ GABAergic interneurons can be identified by monitoring GFP fluorescence. Congruous with the observed loss of *Nkx2.1*‐derived HCF‐1‐negative cells, there was a significant reduction in GAD67‐GFP^+^‐interneurons in the cortex (by 38%) of *Nkx2.1‐Cre^+^*; *Gad1‐GFP^+^*; *Hcfc1^lox/Y^* knockout brains compared to control *Nkx2.1‐Cre^+^*; *Gad1‐GFP* ^+^; *Hcfc1^+/Y^* brains (compare Supp. Fig. 4A to B; see quantitation Supp. Fig. 4G). A similar reduction in GABAergic interneurons was also seen in the corpus callosum (by 49%) of *Nkx2.1‐Cre^+^*; *Gad1‐GFP^+^*; *Hcfc1^lox/Y^* knockout brains (compare Supp. Fig. 4C to D; see quantitation Supp. Fig. 4G). Hence, a large percentage of HCF‐1‐negative GABAergic interneurons generated from the Nkx2.1‐positive ventral telencephalic region apparently fails to reach their target regions.

**Figure 7:**
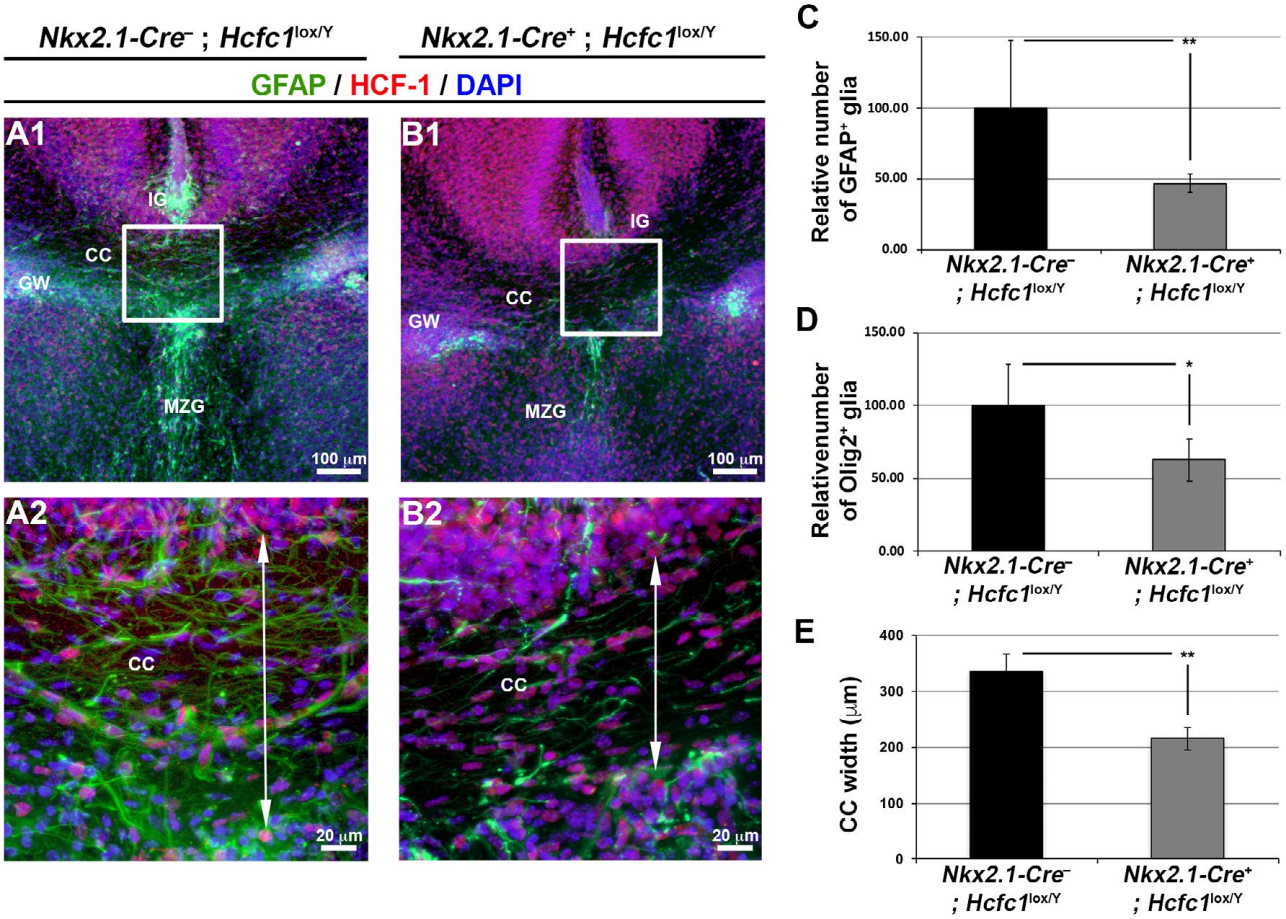
*Nkx2.1‐Cre* mediated loss of HCF‐1 leads to decreased presence of glial cells and reduced corpus callosum width in the postnatal brains. (A‐B) Immunofluorescence analysis of cryo‐sections from *Nkx2.1‐Cre^–^*; *Hcfc1^lox/Y^* (A) and *Nkx2.1‐Cre^+^*; *Hcfc1^lox/Y^* (B) male brains at P1 stained with DAPI (blue) and antibodies against GFAP (green) and HCF‐1 (red). The boxed region in A1 and B1 is shown at higher magnification in A2 and B2, respectively. The two‐sided arrow line in A2 and B2 depicts the thickness of the corpus callosum (CC). IG, indusium griseum; GW, glial wedge; MZG, midline zipper glia. Scale bars are indicated in the figure. (C) Graph showing the relative percentages of GFAP^+^‐glia within the CC in control *Nkx2.1‐Cre^–^*; *Hcfc1^lox/Y^* (n = 2) and knockout *Nkx2.1‐Cre^+^*; *Hcfc1^lox/Y^* (n = 3) male brains at P1. The number of GFAP^+^‐glia within the CC of *Nkx2.1‐Cre^–^*; *Hcfc1^lox/Y^* control brains was calculated, and the mean value was set as 100%. The percentage of GFAP^+^‐glia within the CC of *Nkx2.1‐Cre^+^*; *Hcfc1^lox^* ^/Y^ mutant brains was then calculated relative to the value in control sections. The difference between relative percentages of GFAP^+^‐glia within the CC in control *Nkx2.1‐Cre^–^*; *Hcfc1^lox/Y^* and knockout *Nkx2.1‐Cre^+^*; *Hcfc1^lox/Y^* male brains was significant (*P*‐value 0.005). (D) Graph showing the relative percentages of Olig2^+^‐cells within the CC in control *Nkx2.1‐Cre^–^*; *Hcfc1^lox/Y^* (n = 3) and knockout *Nkx2.1‐Cre^+^*; *Hcfc1^lox/Y^* (n = 3) male brains at P1. The number of Olig2^+^‐cells within the CC of *Nkx2.1‐Cre^–^*; *Hcfc1^lox/Y^* control brains was calculated, and the mean value was set as 100%. The percentage of Olig2^+^‐cells within the CC of *Nkx2.1‐Cre^+^*; *Hcfc1^lox/Y^* mutant brains was then calculated relative to the value in control sections. The difference between relative percentages of Olig2^+^‐cells within the CC in control *Nkx2.1‐Cre^–^*; *Hcfc1^lox/Y^* and knockout *Nkx2.1‐Cre^+^*; *Hcfc1^lox/Y^* male brains was significant (*P*‐value 0.047). (E) Graph showing the width of corpus callosum (CC) of control *Nkx2.1‐Cre^–^*; *Hcfc1^lox/Y^* (n = 3) and knockout *Nkx2.1‐Cre* ^+^; *Hcfc1^lox/Y^* (n = 3) brains at P0. The difference between the width of corpus callosum (CC) of control *Nkx2.1‐Cre^–^*; *Hcfc1^lox/Y^* and knockout *Nkx2.1‐Cre* ^+^; *Hcfc1^lox/Y^* brains was significant (*P*‐value 0.007).

Secondly, to identify *Nkx2.1*‐derived glia, which populate the corpus callosum and its surrounding regions (Minocha *et al.*, 2017), we probed the astroglia and oligodendrocytes. We assayed the effect of loss of HCF‐1 on *Nkx2.1*‐derived astroglia by immunostaining against GFAP, a glial marker, in the *Nkx2.1‐Cre^+^*; *Hcfc1^lox/Y^* brains. Indeed, as shown in Figure 7, a reduction in the number of GFAP‐positive astroglia was seen in the corpus callosum (CC), indusium griseum (IG), midline zipper glia (MZG), and glial sling (GS) of the *Nkx2.1‐Cre^+^*; *Hcfc1^lox/Y^* knockout brains when compared to control *Nkx2.1‐Cre^–^*; *Hcfc1^lox/Y^* brains (compare Fig. 7A to B). Quantification revealed a loss of approximately 50% GFAP‐positive glia within the CC region (Fig. 7C). As evidenced by Olig2 immunofluorescence, the *Nkx2.1*‐derived oligodendrocytes were also reduced in *Nkx2.1‐Cre^+^*; *Hcfc1^lox/Y^* knockout brains when compared to control *Nkx2.1‐Cre^–^*; *Hcfc1^lox/Y^* brains within the corpus callosum (Fig. 7D). Hence, the generation of *Nkx2.1*‐derived glia, both astroglia and oligodendrocytes, is severely affected due to the loss of HCF‐1.

Similar results were obtained upon using a temporally activatable Cre‐recombinase transgene called *GLAST‐CreERT2*, which is primarily expressed in astroglia (Mori *et al.*, 2006). We compared the effects of tamoxifen treatment of *GLAST‐CreERT2^+^*; *Hcfc1^lox/Y^* and *GLAST‐CreERT2^–^*; *Hcfc1^lox/Y^* mice at E16.5 on astroglia development. We observed a reduction in the number of GFAP‐positive astroglia in both the corpus callosum (compare Supp. Fig. 5A to B) and anterior commissure region (compare Supp. Fig. 5C to D). Quantification revealed a loss of approximately 50% GFAP‐positive glia within the corpus callosum and 40% GFAP‐positive glia within the anterior commissure region (Supp. Fig. 5E). Thus, these results also suggest a role of HCF‐1 in brain astroglia development.

### Loss of HCF‐1 Causes Corpus Callosum and Anterior Commissure Defects

Here, we investigated the effects of the loss of *Nkx2.1*‐lineage cells upon the absence of HCF‐1 on brain development. In the analysis of the disappearance of *Nkx2.1*‐lineage cells in *Nkx2.1‐Cre^+^*; *Rosa‐GFP^+^*; *Hcfc1^lox/Y^* brains shown in Figure 6, we noted a number of morphological defects, particularly commissural and cortical. We first investigated the commissural and then the cortical defects, and used in each case glial‐ and neuronal‐specific immunofluorescence markers to follow the contribution of glia and neurons to the defective structures observed. To avoid a conflicting GFP fluorescence signal from the *Rosa‐GFP* transgene, it was excluded from the mouse strains used in the experiments described below.

Relative to the commissures, we observed mild and strong defects in the corpus callosum and anterior commissure regions, respectively. The corpus callosum was clearly thinner but otherwise appeared normal in *Nkx2.1‐Cre^+^*; *Hcfc1^lox/Y^* knockout brains (compare Fig. 7A to B; Fig. 7E). In contrast, the anterior commissure exhibited gross distortions, which is consistent with the finding that selective ablation of *Nkx2.1*‐lineage post‐mitotic cells leads to deflection of anterior commissure axons from their normal trajectory and improper formation of the anterior commissure (Minocha *et al.*, 2015b).

**Figure 8:**
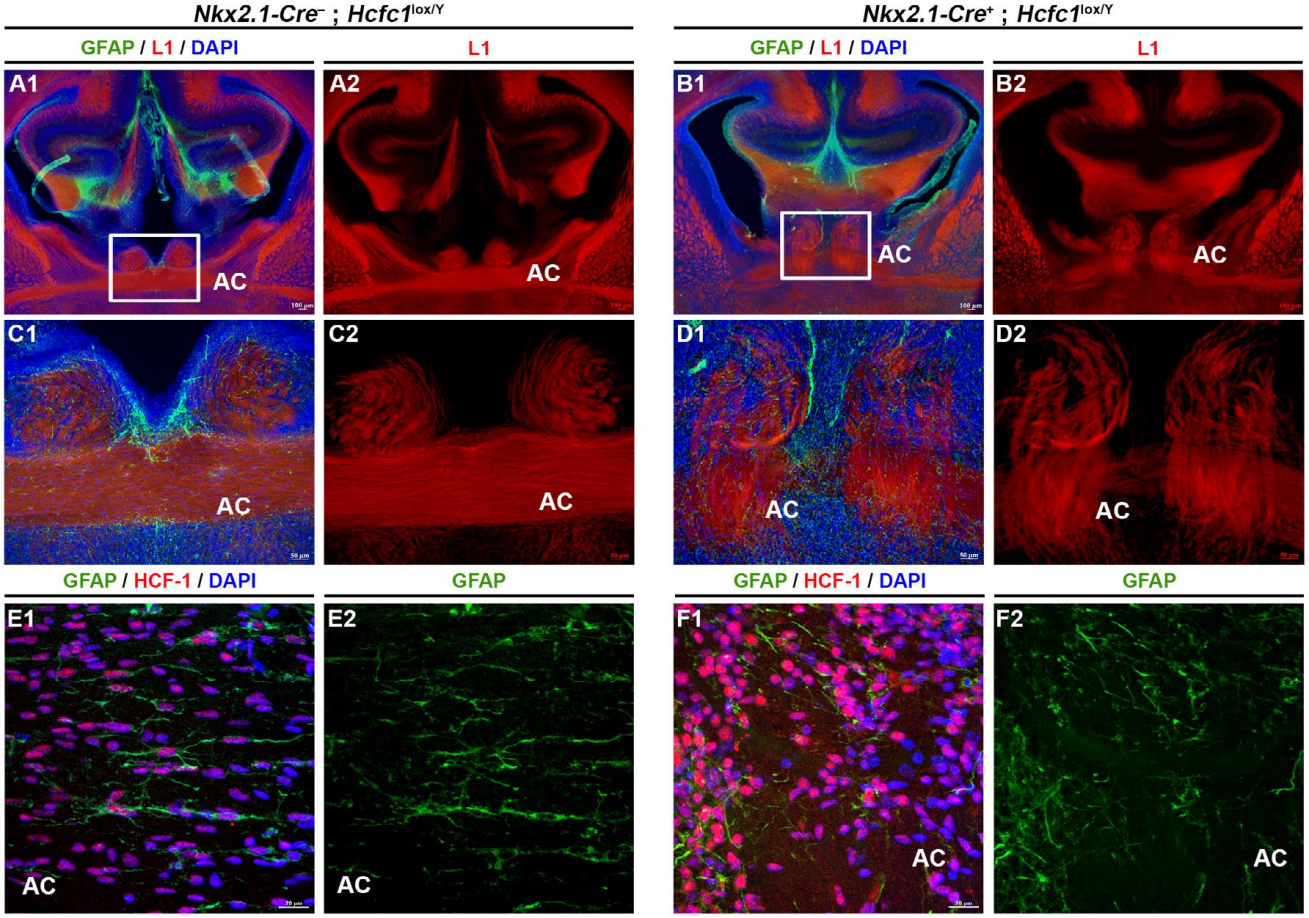
*Nkx2.1‐Cre* mediated loss of HCF‐1 causes defects in proper formation of anterior commissure. (A‐D) Immunofluorescence analysis of cryo‐sections from control *Nkx2.1‐Cre* ^–^; *Hcfc1^lox/Y^* (A and C) and knockout *Nkx2.1‐Cre* ^+^; *Hcfc1^lox/Y^* (B and D) embryonic brains at E18.5 stained with DAPI (blue) and antibodies against astroglial marker, glial fibrillary acidic protein (GFAP; green) and axonal marker, L1 cell adhesion molecule (red). The boxed region in A1 and B1 is shown at higher magnification in C and D, respectively. Co‐labeling with DAPI, GFAP, and L1 is shown in A1, B1, C1, and D1, whereas labeling with only L1 is shown in A2, B2, C2, and D2. (E–F) Immunofluorescence analysis of cryo‐sections from control *Nkx2.1‐Cre* ^–^; *Hcfc1^lox/Y^* (E) and knockout *Nkx2.1‐Cre* ^+^; *Hcfc1^lox/Y^* (F) embryonic brains at E18.5 stained with DAPI (blue) and antibodies against GFAP (green) and HCF‐1 (red). Co‐labeling with DAPI, GFAP, and HCF‐1 is shown in E1 and F1, whereas labeling with only GFAP is shown in E2 and F2. AC, anterior commissure. Scale bars are indicated in the figure.

Indeed, serial sections of the *Nkx2.1‐Cre^+^*; *Hcfc1^lox/Y^* knockout male brains (Supp. Fig. 6) indicated that the anterior commissure is not properly formed (see red arrows). Labeling of the anterior commissure axons with the axonal L1 cell adhesion molecule (L1CAM) marker displayed dissimilar trajectories in control *Nkx2.1‐Cre^–^*; *Hcfc1^lox/Y^* versus *Nkx2.1‐Cre^+^*; *Hcfc1^lox/Y^* knockout brains (Fig. 8A–D). Though anterior commissure axons were found near the midline in *Nkx2.1‐Cre^+^*; *Hcfc1^lox/Y^* knockout brains, only some axons successfully crossed the midline while most seemed to be stalled (compare Fig. 8A and C to B and D). Furthermore, the anterior commissure axons were loosely bundled and adopted abnormal trajectories in *Nkx2.1‐Cre^+^*; *Hcfc1^lox/Y^* knockout brains compared to the tightly bundled axons of the anterior commissure in control *Nkx2.1‐Cre^–^*; *Hcfc1^lox/Y^* brains (compare Fig. 8C to D). These defects may be explained by the greatly reduced and disorganized status of Nkx2.1‐lineage cells in the anterior commissure and its surrounding regions seen in the *Nkx2.1‐Cre^+^*; *Rosa‐GFP^+^*; *Hcfc1^lox/Y^* knockout versus normal *Nkx2.1‐Cre^+^*; *Rosa‐GFP^+^*; *Hcfc1^+/Y^* brains (compare Fig. 6E to F).

As the *Nkx2.1*‐lineage population comprises both neurons and glia, we assayed the organization of these two populations in the anterior commissure region of *Nkx2.1‐Cre^+^*; *Hcfc1^lox/Y^* knockout brains. The GABAergic interneurons (compare Supp. Fig. 4E to F) and the GFAP‐positive glia (compare Fig. 8E to F) were both disorganized in the anterior commissure of *Nkx2.1‐Cre^+^*; *Gad1‐GFP^+^*; *Hcfc1^lox/Y^* knockout brains and its surrounding regions compared to the control *Nkx2.1‐Cre^+^*; *Gad1‐GFP* ^+^; *Hcfc1^+/Y^* brains*.*

Hence, loss of HCF‐1 in *Nkx2.1*‐lineage cells affects their survival and as a result midline commissures, particularly the anterior commissure, are malformed in mutant brains.

### Early Loss of HCF‐1 Causes Cortical Defects

Nkx2.1 plays a key role in the specification and production of GABAergic interneurons and glia from the ventral telencephalon that migrate into the striatum and cerebral cortex (Sussel *et al.*, 1999; Anderson *et al.*, 2001; Corbin *et al.*, 2001; Marin and Rubenstein, 2001; Kessaris *et al.*, 2006; Minocha *et al.*, 2015a; 2015b). To elucidate the effect on cortical development of loss of migratory neurons and glia upon *Hcfc1*‐gene disruption in *Nkx2.1‐Cre^+^*; *Hcfc1^lox/Y^* knockout brains, we analyzed P0‐to‐P1 mice.

**Figure 9:**
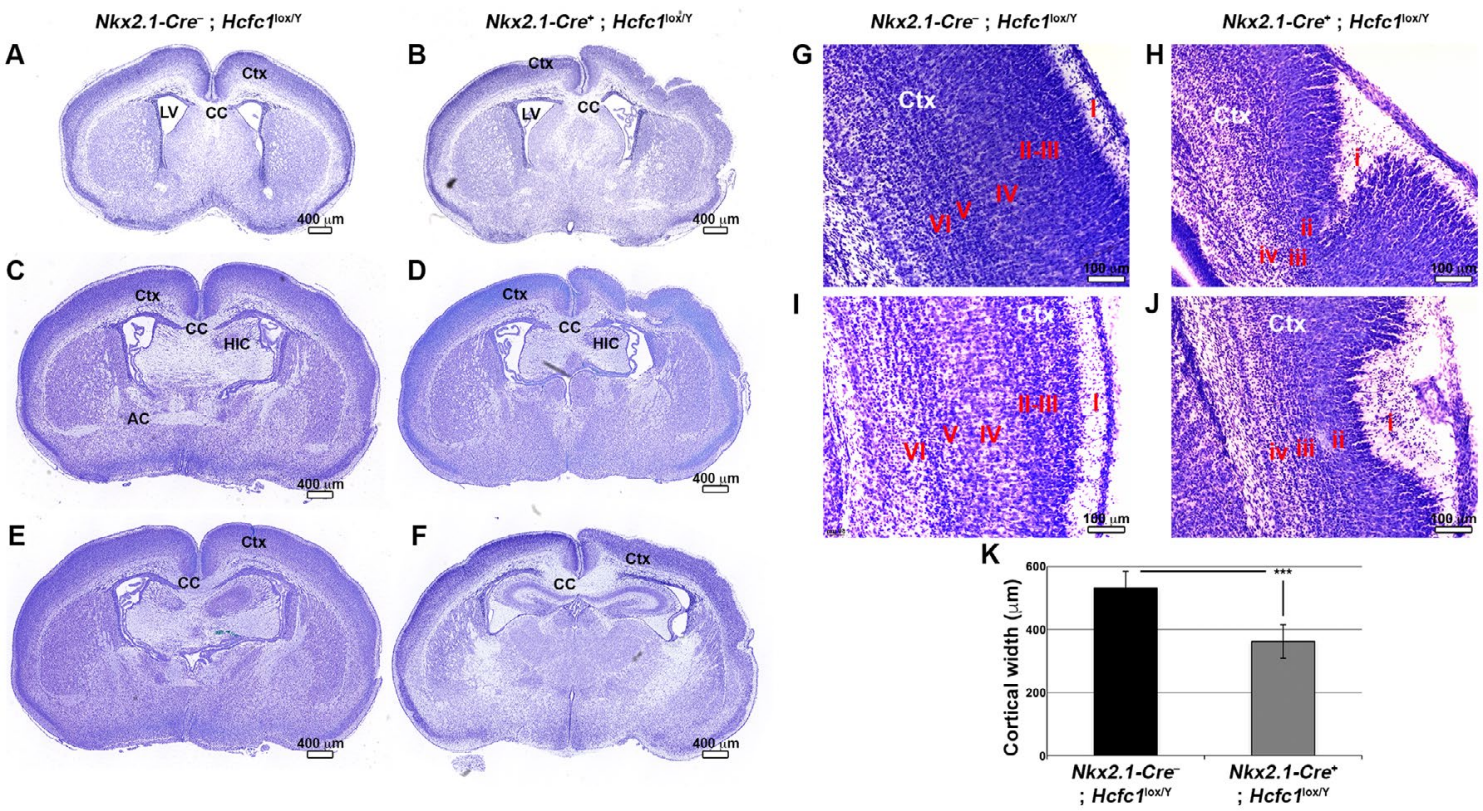
Nkx2.1‐Cre mediated loss of HCF‐1 causes severe cortical defects. Nissl staining of paraffin‐embedded sections from control *Nkx2.1‐Cre^–^*; *Hcfc1^lox/Y^* (A, C, E, G, and I) and knockout *Nkx2.1‐Cre^+^*; *Hcfc1^lox/Y^* (B, D, F, H, and J) male brains at P1. (A–F) Three representative coronal sections arranged from rostral‐to‐caudal poles of the control *Nkx2.1‐Cre^–^*; *Hcfc1^lox/Y^* (A, C, and E) and knockout *Nkx2.1‐Cre^+^*; *Hcfc1^lox/Y^* (B, D, and F) male brains are shown. (G–J) Higher magnification views of the cortical region from *Nkx2.1‐Cre^–^*; *Hcfc1^lox/Y^* (G and I) and *Nkx2.1‐Cre^+^*; *Hcfc1^lox/Y^* (H and J) male brains are shown. Cortical layers from I‐to‐VI are indicated in the control *Nkx2.1‐Cre^–^*; *Hcfc1^lox/Y^* brains. Only four clearly discernible layers i‐to‐iv are labeled in knockout *Nkx2.1‐Cre^+^*; *Hcfc1^lox/Y^* brains. AC, anterior commissure; CC, corpus callosum; Ctx, cortex; HIC, hippocampal commissure; LV, lateral ventricle. Scale bars are indicated in the figure. (K) Graph showing the cortical thickness of control *Nkx2.1‐Cre^–^*; *Hcfc1^lox/Y^* (n = 9) and knockout *Nkx2.1‐Cre* ^+^; *Hcfc1^lox/Y^* (n = 21) brains at P0. The difference between cortical thickness in control *Nkx2.1‐Cre^–^*; *Hcfc1^lox/Y^* and knockout *Nkx2.1‐Cre* ^+^; *Hcfc1^lox/Y^* brains was highly significant (*P*‐value 6.65 × 10^−7^).

Although less frequent than the commissure defects, cortical defects were still common. Such defects are clearly visible in both hemispheres in the *Nkx2.1‐Cre^+^*; *Hcfc1^lox/Y^* knockout brain serial sectioning shown in Supplemental Figure 6 (black arrows). Indeed, we did not observe a preference for defects in one or the other hemisphere. Eleven of 19 analyzed *Nkx2.1‐Cre^+^*; *Hcfc1^lox/Y^* knockout brains exhibited severe cortical defects in at least one hemisphere (see Fig. 9B and D compared to A and C) whereas the rest exhibited milder cortical defects (see Fig. 9F compared to E) and yet the cortices were thinner when compared to control embryos (Fig. 9K). In comparison to the unaltered six‐layered cortex of control *Nkx2.1‐Cre^–^*; *Hcfc1^lox/Y^* brains (Fig. 9G and I), the laminar organization of the *Nkx2.1‐Cre^+^*; *Hcfc1^lox/Y^* knockout brain cortices (Fig. 9H and J) was disturbed, perhaps missing layers.

**Figure 10:**
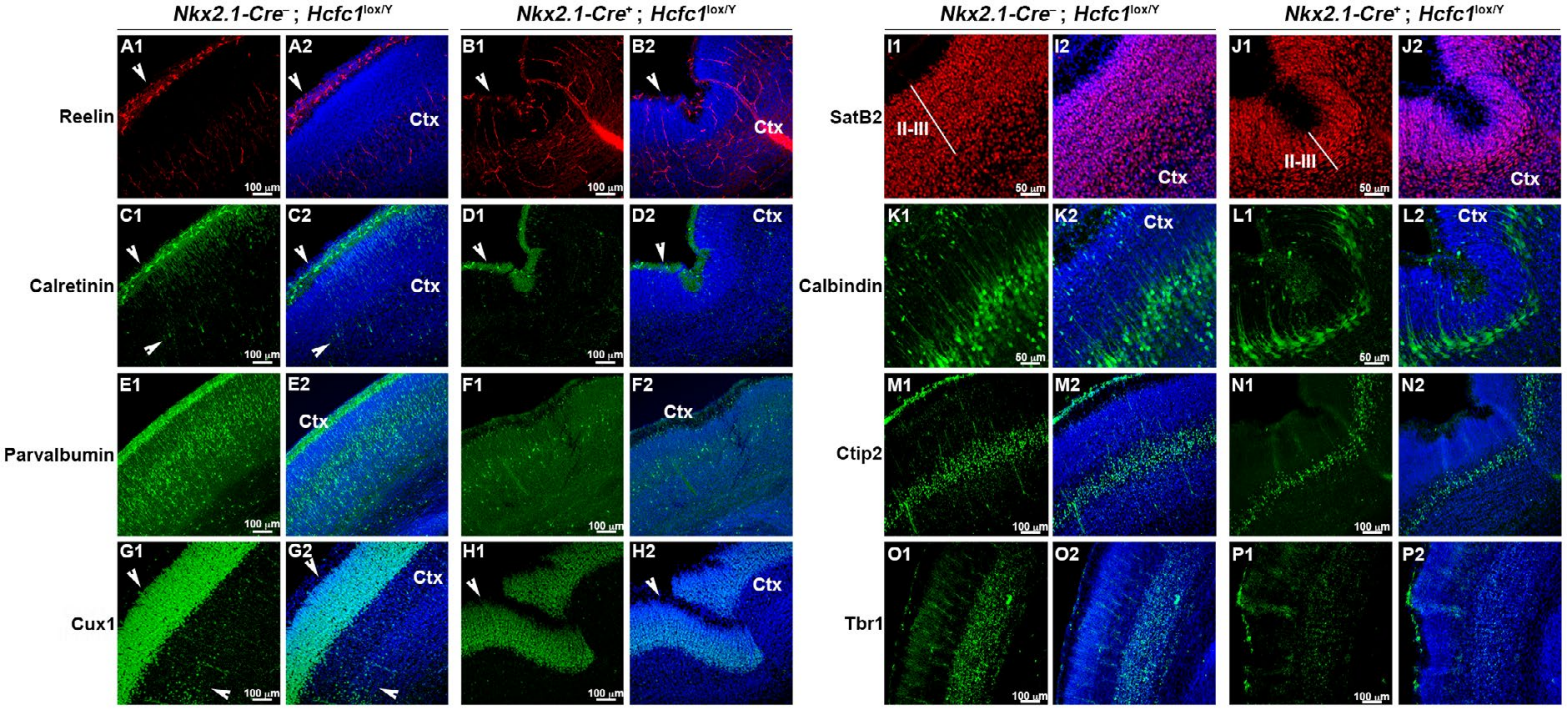
*Nkx2.1‐Cre* mediated loss of HCF‐1 causes alterations in cortical lamination. Immunofluorescence analysis of cryo‐sections from control *Nkx2.1‐Cre* ^–^; *Hcfc1^lox/Y^* (A, C, E, G, I, K, and M) and knockout *Nkx2.1‐Cre* ^+^; *Hcfc1^lox/Y^* (B, D, F, H, J, L, and N) brains at P1 stained with DAPI (blue) and antibodies against cortical neuronal markers, namely Reelin (red; A and B); Calretinin (green; C and D); Parvalbumin (green; E and F); Cux1 (green; G and H); SatB2 (red; I and J); Calbindin (green; K and L); Ctip2 (green; M and N); and Tbr1 (green; O and P). Left‐side panels show staining with only the labeled marker (A1, B1, C1, D1, E1, F1, G1, H1, I1, J1, K1, L1, M1, N1, O1, and P1), whereas the right‐side panels show co‐staining of labeled marker and DAPI (A2, B2, C2, D2, E2, F2, G2, H2, I2, J2, K2, L2, M2, N2, O2, and P2). The arrowheads in A and B point to Reelin‐positive cells. The arrowheads in C and D point to Calretinin‐positive cells. The arrowheads in G and H point to Cux1‐positive cells. Ctx, cortex. Scale bars are indicated in the figure.

To probe for layering defects, we performed immunostaining for the cortical markers listed in Figure 10. We used markers Reelin (Fig. 10A,B) and Calretinin (Fig. 10C,D) for layer I; Calretinin (Fig. 10C,D), Parvalbumin (Fig. 10E,F), Cux1 (Fig. 10G,H), SatB2 (Fig. 10I,J), and Calbindin (Fig. 10K,L) for layers II‐III; Parvalbumin (Fig. 10E,F) and Cux1 (Fig. 10G,H) for layer IV; Parvalbumin (Fig. 10E,F), Calbindin (Fig. 10K,L), and Ctip2 (Fig. 10M,N) for layer V; and Parvalbumin (Fig. 10E,F) and Tbr1 (Fig. 10O,P) for layer VI. From the cortical layer analysis and NISSL staining (Fig. 9), it appeared that layer I though deformed is present, whereas layers II, III, and V though present are relatively thinner in *Nkx2.1‐Cre^+^*; *Hcfc1^lox/Y^* knockout brains when compared to control brains. Interestingly, it appeared that the layer IV and layer VI are either much thinner or absent.

Although *Nkx2.1‐Cre^+^*; *Hcfc1^lox/Y^* knockout brains exhibit these malformations, the cells within the cortex are essentially all HCF‐1 positive and are proliferating as found in normal brains (Supp. Fig. 7). Thus, it is likely that it is the absent *Nkx2.1*‐lineage cells that are causing the cortical aberrations. These aberrations resemble those that occur in asymmetric polymicrogyria, which are typically characterized by the presence of irregular cortical folds and a reduced number of cortical layers (Barkovich, 2010; Guerrini and Parrini, 2010). Such malformations could be one way by which human patients carrying *HCFC1* mutations display intellectual disability.

## Discussion

We have shown that conditional loss of HCF‐1 in *Nkx2.1*‐derived neurons and glia leads to commissural defects affecting primarily the AC as well as asymmetric polymicrogyria‐like cortical defects. These defects appear to arise because of a reduced number of embryonic *Nkx2.1*‐derived neurons and glia owing to their increased cell death. This reduced presence of *Nkx2.1*‐derived cells leads to formation of severely malformed AC whose axons deviate from the normal path, being deflected both dorsally and ventrally. Also, the laminar organization of the cortex is locally disturbed generating irregular folds where cortical layers are reduced in number and thickness.

### The Activity of HCF‐1 Differs Depending on Cell Context

HCF‐1 is a transcriptional co‐regulator and is important for several aspects of the cell cycle in tissue culture cells and during liver regeneration (Goto *et al.*, 1997; Reilly and Herr, 2002; Julien and Herr, 2003; Minocha *et al.*, 2016a; 2016b). Interestingly, loss of HCF‐1 does not seem to affect the proliferation capacity of Nkx2.1‐positive precursor cells in the ventral telencephalic region. These results are consistent with a previous report where knockdown of HCF‐1 was shown to display increased proliferation of neural precursors in an *in vitro* neurosphere assay (Jolly *et al.*, 2015). Our results show that, instead of inhibiting cell proliferation, loss of HCF‐1 leads to increased cell death of *Nkx2.1*‐derived post‐mitotic cells (GABAergic interneurons and glia) in the ventral telencephalic region. These results demonstrate that the role of HCF‐1, likely as a transcriptional regulator of many genes, can differ depending on the cell context. This finding of differing roles of HCF‐1 in different cell contexts complements that in the context of resting adult mouse hepatocytes where loss of HCF‐1 leads to hepatocyte malfunction (Minocha *et al.*, 2018). HCF‐1 appears to have evolved to play a multitude of cell‐specific roles in the regulation of gene expression, probably principally gene transcription but also through protein stabilization as in the case of PGC1α in hepatocytes (Ruan *et al.*, 2012; Minocha *et al.*, 2018). In this manner, it serves as a broad and versatile potentiator of cell function.

### Effects of HCF‐1 Loss in Nkx2.1‐lineage Cells on Embryonic Brain Development

*Nkx2.1*‐derived cells (GABAergic interneurons and glia) have the striking capacity to migrate to numerous areas of the brain including the commissures and cortex after their initial formation primarily in the MGE and POA. Here, owing to their death, *Nkx2.1*‐derived cells cannot migrate apparently causing *Nkx2.1‐Cre^+^*; *Hcfc1^lox/Y^* knockout mice to display the numerous diverse brain defects observed.

These results are consistent with previous reports where it has been shown that polymicrogyria primarily develops due to either reduced proliferation of neural precursors, disturbed neuronal migration or aberrant cortical organization (Guerrini and Filippi, 2005; Guerrini and Parrini, 2010). A role of GABAergic interneurons in cortical development has also been shown in *Arx*‐deficient mice that recapitulate features of cortical malformation called as X‐linked Lissencephaly with absent corpus callosum and Ambiguous Genitalia (XLAG) in humans (Bonneau *et al.*, 2002; Kitamura *et al.*, 2002). XLAG is characterized by agenesis of CC, poorly laminated cortex, microcephaly, and epilepsy (Bonneau *et al.*, 2002). Together, these results all point to the importance of GABAergic interneurons in cortical development.

Cases of polymicrogyria are known to occur sporadically, though several families have also been observed with loci mapping to the X chromosome, including Xq28 (Geerdink *et al.*, 2002; Villard *et al.*, 2002; Barkovich, 2010). In humans, *HCFC1* resides on Xq28 (Frattini *et al.*, 1994; Wilson *et al.*, 1995) and has been strongly implicated in development of intellectual disability (Huang *et al.*, 2012; Yu *et al.*, 2013; Jolly *et al.*, 2015). Intellectual disability is often a clinical manifestation of cortical malformations such as polymicrogyria. HCF‐1 is essential for survival of *Nkx2.1*‐lineage cells, GABAergic interneurons and glia, whose absence in turn causes cortical defects strongly resembling polymicrogyria. We suggest that these cortical malformations observed upon loss of HCF‐1 in subpopulations of GABAergic interneurons and glia may well also be present in human intellectual disability patients carrying *HCFC1* mutations.

### Uncovering a New Role of Nkx2.1‐Lineage Cells in Polymicrogyria

The effect of Nkx2.1‐Cre‐induced loss of HCF‐1 on anterior commissure formation closely mimics those observed upon ablation of *Nkx2.1*‐derived cells, both neurons and glia, with the help of diphtheria toxin (Minocha *et al.*, 2015b). But, interestingly, ablation of *Nkx2.1*‐derived cells did not show any cortical aberrations (Minocha *et al.*, 2015b), such as those visible in brains lacking HCF‐1 in *Nkx2.1*‐derived cells. These previous studies involving ablation of *Nkx2.1*‐derived cells failed to observe an effect on cortical development apparently due to the delayed accumulation of diphtheria toxin where precursors are not affected and also because embryos died before birth probably owing to an effect on lung development. In our study, as we were able to analyze the brains of neonatal pups lacking HCF‐1 in *Nkx2.1*‐derived cells, we could observe the cortical aberrations. Hence, our study uncovers a new role of *Nkx2.1*‐derived cells during cortical development.

## Author Contributions

The experiments were conceived and designed by S.M. and W.H. The experiments were performed by S.M. S.M. and W.H. analyzed the data and prepared the manuscript. Both authors participated in the discussion of the data and in production of the final version of the manuscript.

## Conflict of interest

The authors declare that there are no conflicts of interest.

## Data Availability

The data that support the findings of this study are available from the corresponding author upon reasonable request.
