# Supplementary figures and images for: Cortical and Commissural Defects Upon HCF‐1 Loss in Nkx2.1‐Derived Embryonic Neurons and Glia
Source: Dev Neurobiol. 2019 Jun 25;79(6):578–95. doi: 10.1002/dneu.22704 (PMC6771735; doi:10.1002/dneu.22704)

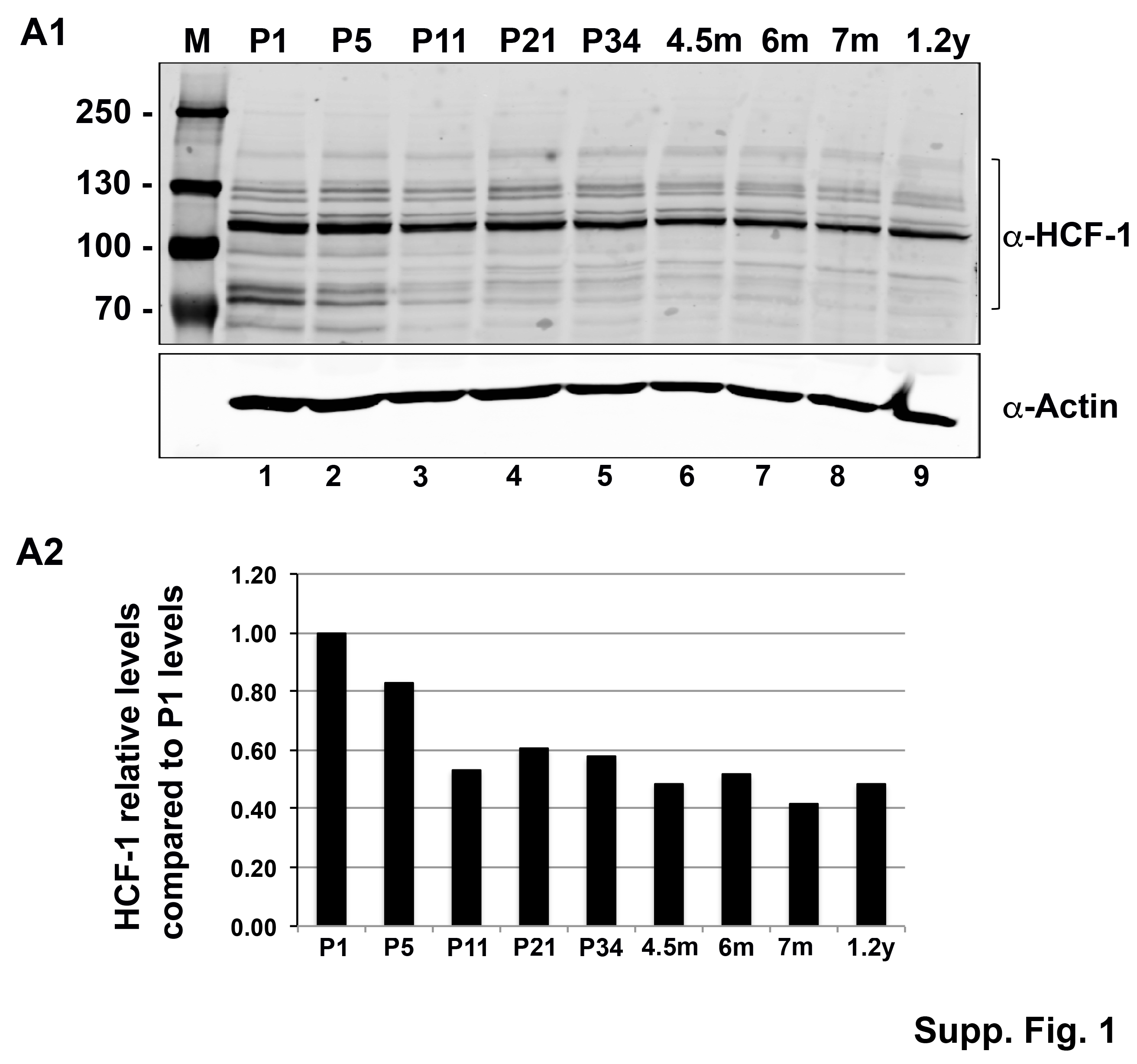

Supplement: Supplementary file 1 [file DNEU-79-578-s001.tif]

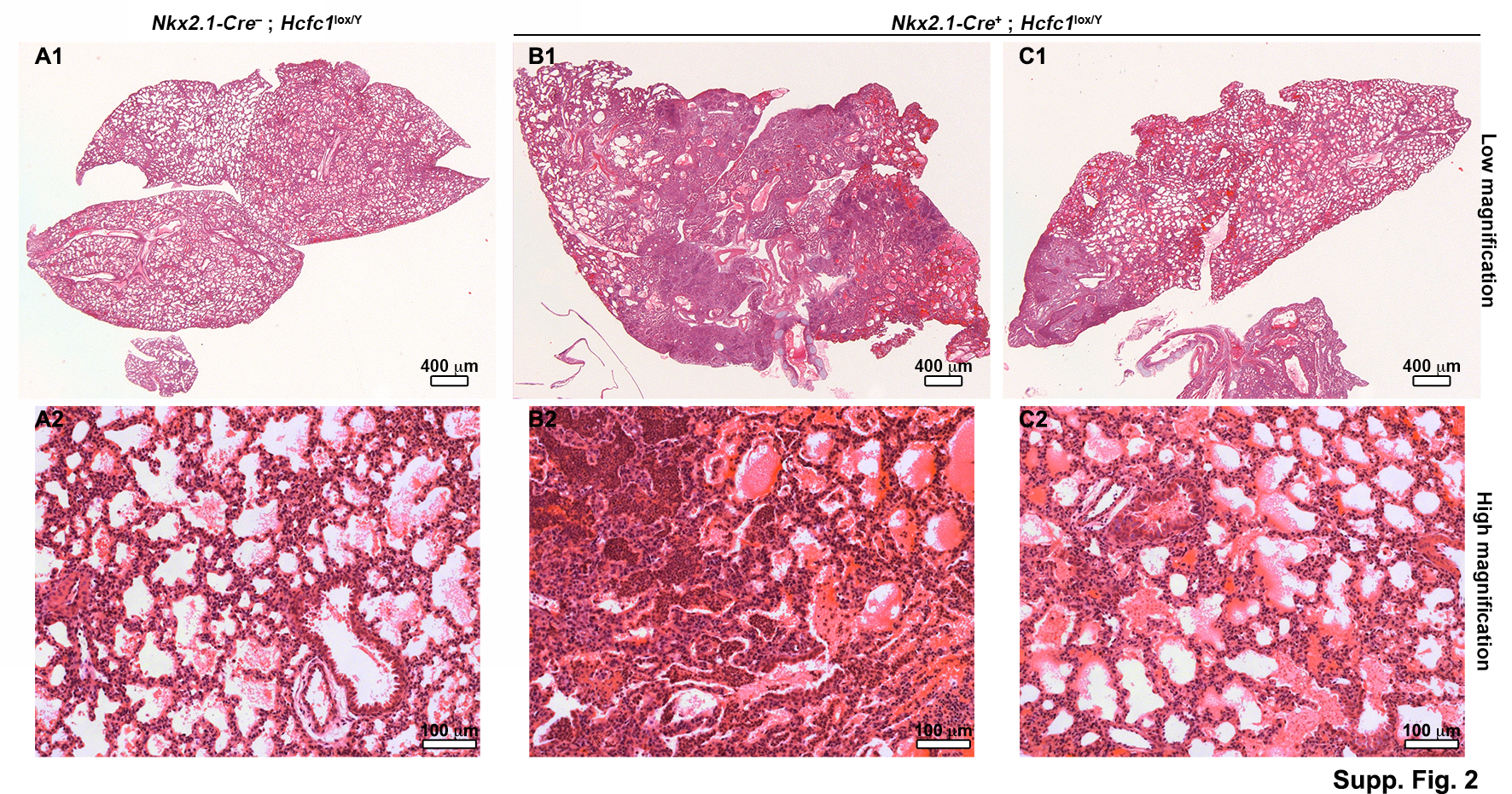

Supplement: Supplementary file 2 [file DNEU-79-578-s002.tif]

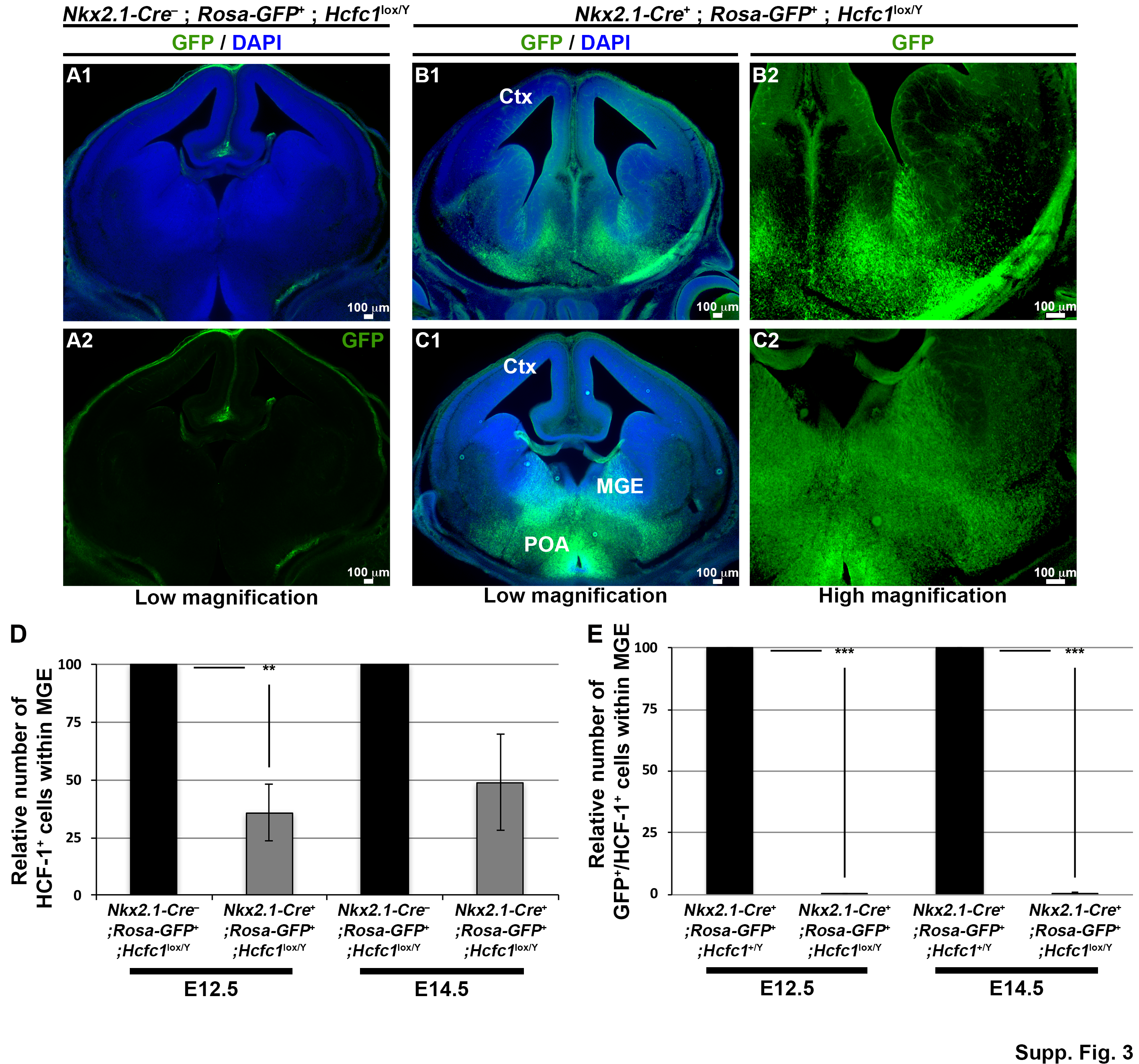

Supplement: Supplementary file 3 [file DNEU-79-578-s003.tif]

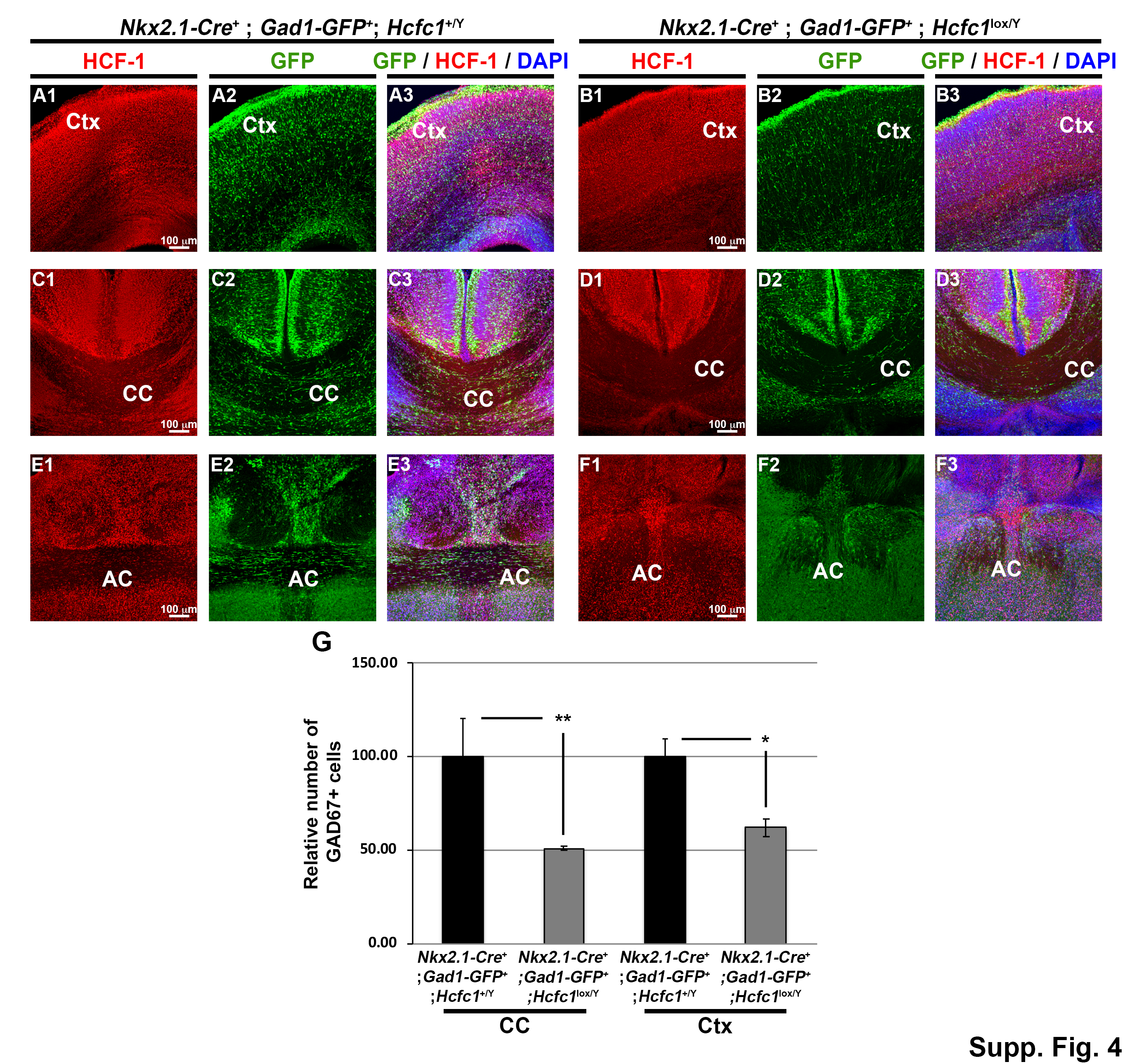

Supplement: Supplementary file 4 [file DNEU-79-578-s004.tif]

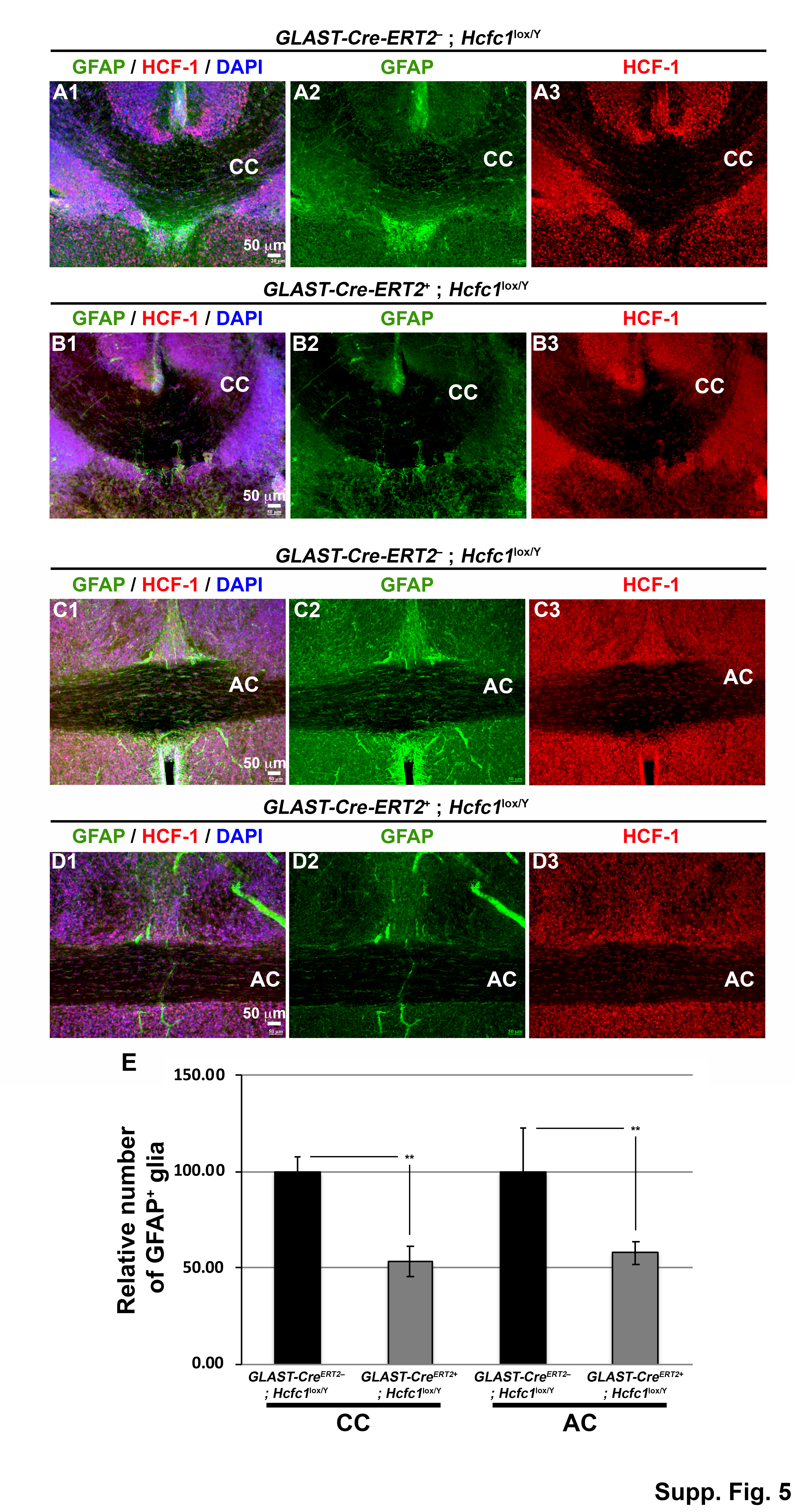

Supplement: Supplementary file 5 [file DNEU-79-578-s005.tif]

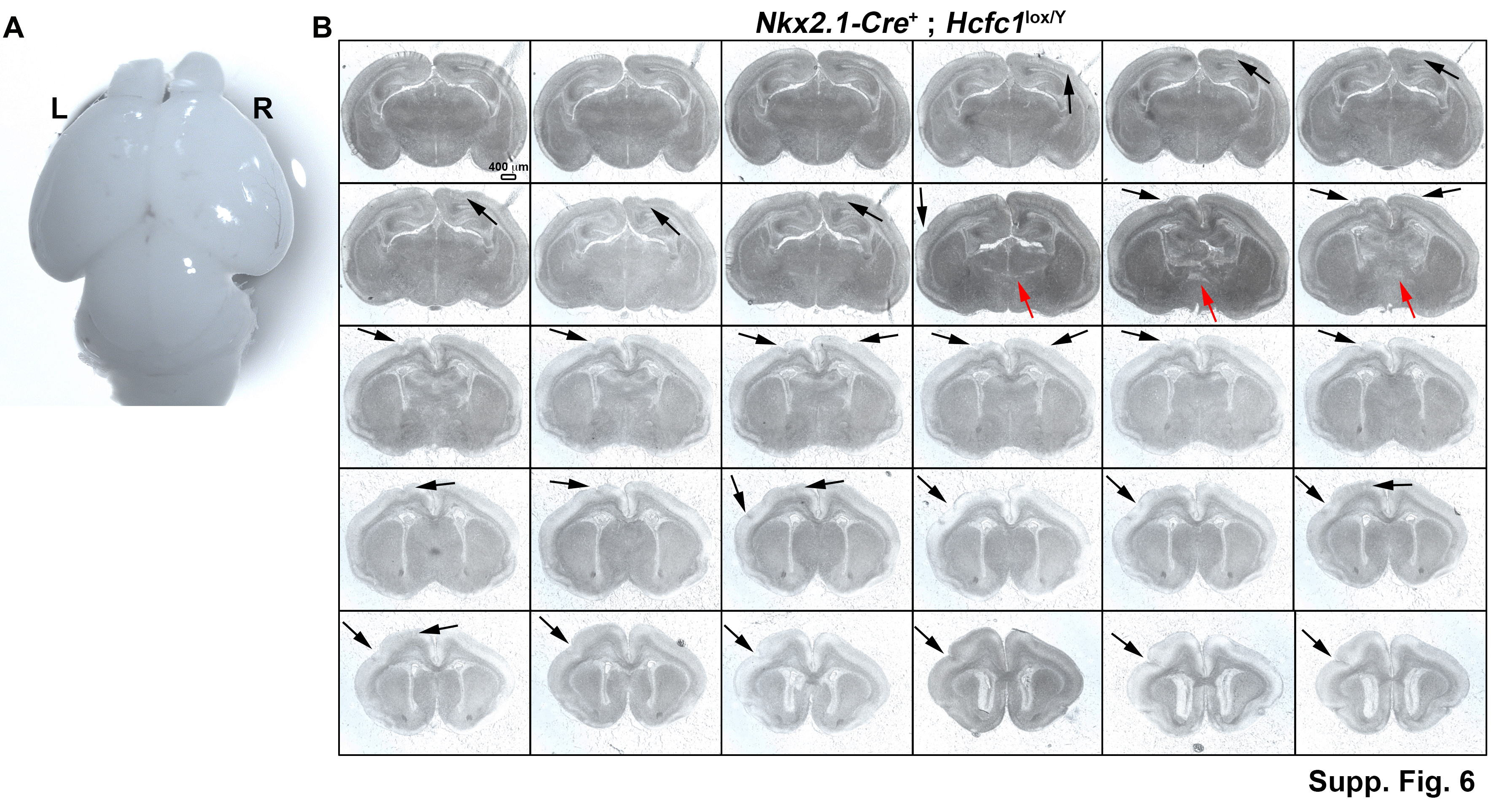

Supplement: Supplementary file 6 [file DNEU-79-578-s006.tif]

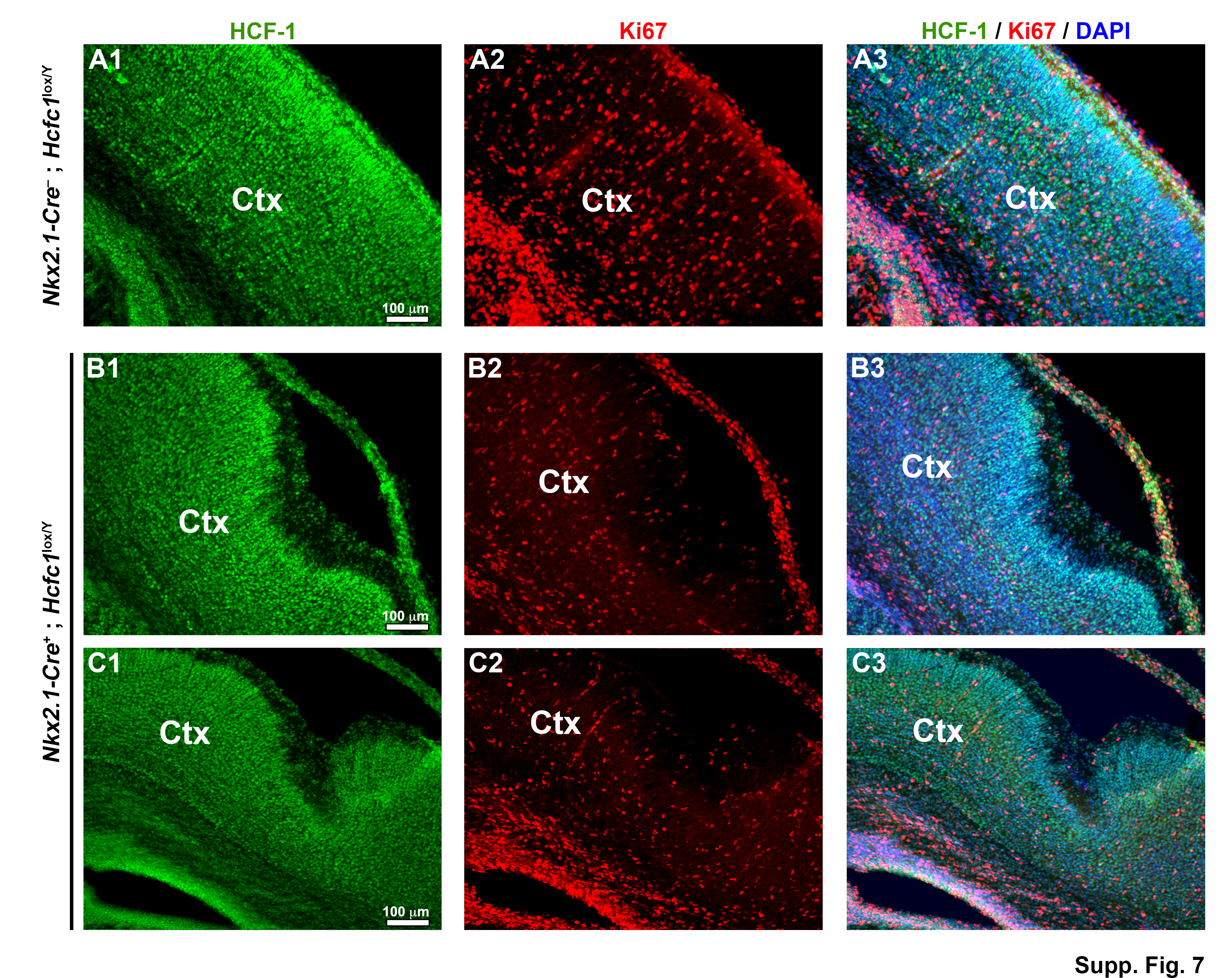

Supplement: Supplementary file 7 [file DNEU-79-578-s007.tif]
